# Western corn rootworm adult activity and immigrant resistance to Bt traits in first-year maize

**DOI:** 10.1371/journal.pone.0325388

**Published:** 2025-06-13

**Authors:** Lance J. Meinke, Jordan D. Reinders, James Clothier, Jeffrey T. Krumm, Clinton D. Pilcher, Matthew W. Carroll, Graham P. Head

**Affiliations:** 1 Department of Entomology, University of Nebraska, Lincoln, Nebraska, United States of America; 2 Department of Statistics, University of Nebraska, Lincoln, Nebraska, United States of America; 3 Midwest Research, Hastings, Nebraska, United States of America; 4 Corteva Agriscience, Johnston, Iowa, United States of America; 5 CropScience Division, Bayer AG, Chesterfield, Missouri, United States of America; University of Tennessee, UNITED STATES OF AMERICA

## Abstract

The western corn rootworm (WCR) *Diabrotica virgifera virgifera* LeConte is an important insect pest of maize (*Zea mays* L.) in the midwestern United States of America (USA) and has evolved resistance to maize hybrids producing toxins from the bacterium *Bacillus thuringiensis* Berliner (Bt). This study was conducted in a landscape with a high proportion of continuous maize (maize planted ≥ two consecutive years) during 2021–2022 in northeast Nebraska, USA to increase our understanding of adult WCR activity in first-year maize and the introduction of Bt resistance by WCR immigrants. Pherocon AM unbaited sticky traps were placed at ear height in first-year maize fields and replaced weekly during adult WCR activity periods to determine density and gender of captured adults. Maize and WCR phenological interactions plus gender-specific behaviors appeared to be key determinants of WCR activity in first-year maize. Comparison of adult emergence and root injury in first- and second-year maize fields confirmed that crop rotation reduces a WCR population to near-zero. Field collections of adults were made from first-year and some adjacent continuous maize fields to estimate Bt susceptibility with Bt bioassays of F_1_ progeny. Similar resistance levels were observed in WCR collections from first-year and many adjacent continuous maize fields. Aggregate study results suggest adjacent maize fields were a major contributor of WCR immigrants. Significant variation in WCR immigration/ colonization and associated Bt resistance levels were observed in first-year maize, so scouting of first-year maize fields is recommended to match appropriate WCR management approaches to relative risk of injury in second-year maize.

## Introduction

The western corn rootworm (WCR), *Diabrotica virgifera virgifera* LeConte (Coleoptera: Chrysomelidae) is an important insect pest of maize, *Zea mays* L., in the United States of America (USA) [1,2]. Several cropping sequences commonly found in the USA Corn Belt can differentially impact WCR population dynamics. Annual rotation of maize with a crop that is not a WCR larval host (i.e., soybean, *Glycine max* (L.) Merr.) is an agronomic practice that will decrease WCR density in first-year maize to near zero, reducing the need for additional WCR control tactics [3,4]. In the western USA Corn Belt, the demand for maize is high for confined livestock operations and ethanol production, leading to higher adoption of continuous maize (consecutive planting of maize for ≥ 2 years) than in eastern USA maize growing areas [2]. However, this agronomic system leads to WCR population build-up over time, making management an annual challenge [5–8]. Feeding injury to maize roots by the WCR larval stage causes most economic loss, which combined with associated management expenditures can annually cost ≥ $2 billion USD in the USA [9].

Transgenic plants which express proteins derived from *Bacillus thuringiensis* Berliner (Bt) that kill WCR larvae when ingested have been widely adopted as the primary component of WCR management programs in continuous maize [10,11]. Initially, single protein Bt hybrids were commercialized (Cry3Bb1 [12], mCry3A [13], Cry34/35Ab1 [14] now reclassified as Gpp34/Tpp35Ab1 [15]; Gpp34/Tpp35Ab1 is used hereafter in the paper). However, after repeated use of single protein hybrids, significant root injury and field-evolved resistance were documented in parts of the USA Corn Belt [16–24]. Widespread levels of resistance in the landscape were eventually recorded over time in Iowa and Nebraska after Bt technologies were introduced [23–27].

To slow the evolution of WCR resistance and mitigate existing resistance, single Bt protein hybrids were phased out and replaced by Bt pyramids (≥ two proteins expressed in a plant targeting a pest) so WCR larvae would have to physiologically overcome more than one toxin to cause control failure in continuous maize (redundant killing [28–30]). A common industry strategy was to include one or more Bt proteins in pyramids that were originally released as single protein hybrids (i.e., Cry3Bb1 x Gpp34/Tpp35Ab1 [31], mCry3A x Gpp34/Tpp35Ab1 [32], mCry3A x eCry3.1Ab [33]. This strategy can be compromized in areas where WCR resistance had previously evolved to one or more Bt components of the pyramid [2,8,11,24].

To extend the durability of current Bt hybrids, there is a need to integrate transgenic maize with existing integrated pest management (IPM) tactics to develop economical and effective integrated resistance management (IRM) programs that delay or mitigate resistance. Crop rotation to a non-host crop is a key component of WCR IRM and IPM strategies [34–36]. The adult WCR exhibits both local dispersal and longer-range migration behaviors [37–39] that can facilitate immigration and recolonization of first-year maize following a crop that does not support larval survival [40]. The WCR densities and Bt susceptibility levels present in WCR populations from the surrounding landscape are key factors that determine rate and level of WCR reinfestation and associated Bt susceptibility in first-year maize. This will also dictate the appropriate management tactics needed in second and third-year maize after crop rotation if a continuous maize agronomic practice is resumed.

The field portion of this study was conducted during 2021–2022 in northeast Nebraska to increase our understanding of WCR activity and introduction of Bt resistance by immigrant WCR in first-year maize in a landscape with a high proportion of continuous maize. In the study areas, continuous maize duration ranged from 2 to > 10 years and was associated with a high concentration of confined livestock. In addition, long-term use of Cry3, Gpp34Ab1/Tpp35Ab1 and Bt pyramided maize hybrids containing Gpp34Ab1/Tpp35Ab1 to manage WCR injury was common and WCR populations exhibited various levels of resistance to Cry3 and Gpp34Ab1/Tpp35Ab1 proteins [8,18,23,24,41]. The specific objectives of this study included: use commercial field case histories to: i) evaluate adult WCR male and female activity patterns in first-year maize following soybean; ii) determine WCR Bt susceptibility of populations collected from first-year maize; and iii) determine recolonization impact on root injury and adult emergence in second-year maize. Results from this study will inform WCR IPM and IRM programs in areas where resistance to Bt traits is common in the landscape.

## Materials and methods

### WCR populations/fields

**Fig 1:**
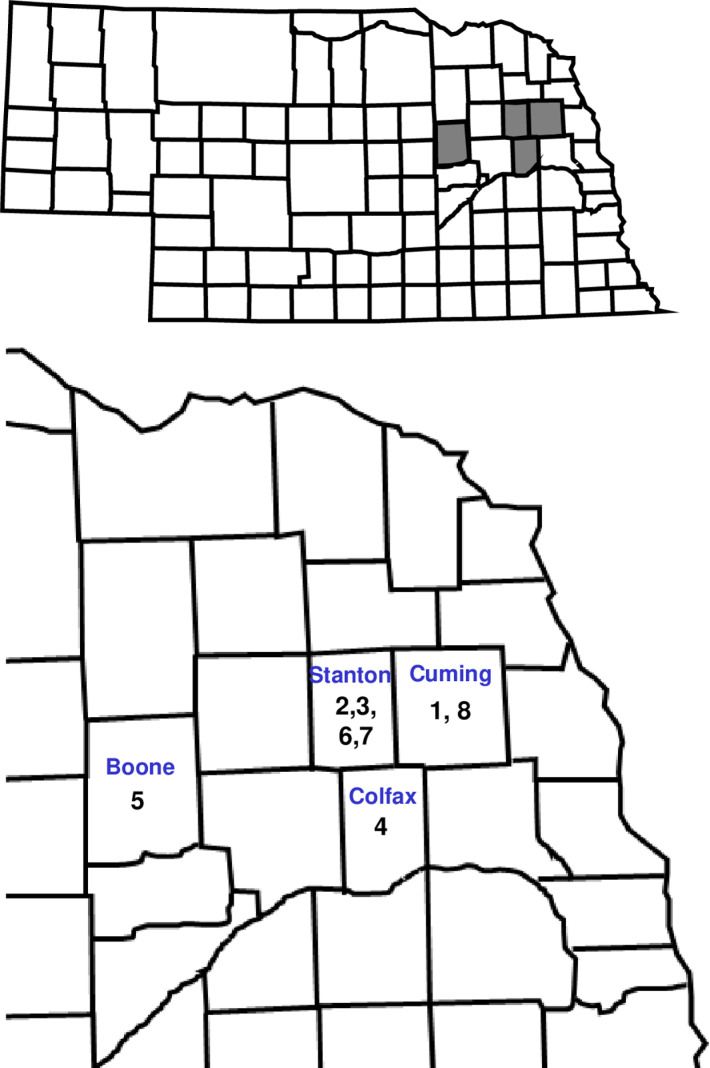
Nebraska state map showing counties in gray where on-farm research was conducted. The expanded map of northeast Nebraska includes the unique numbers assigned to each field/WCR population used in the project. This figure was created by modifying Fig 1 in Meinke et al. PLoS ONE. 2024; 19: e0299483 under the Creative Commons Attribution 4.0 International license.

Farmer cooperators were identified, and permission was granted to conduct this project during 2021–2022 on farms in four counties in northeast Nebraska. A unique number was assigned to each field/WCR population used in the project (Fig 1). All first-year maize fields were randomly selected, were at least 1.6 km apart if in the same county, and followed soybean production the previous year. The phenology of maize fields included in the study was similar to surrounding maize since most maize in each area was planted and emerged at a similar time. General background of each field is presented in Table 1.

**Table 1:** General background of first-year maize fields sampled in 2021-2022.

Year	Field	County	Field Size (Ha)	Sampling Location Within Field	Closest Continuous Maize (m)	Adjacent Landscape^a^
2021	Fd1	Cuming	34.3	SW area	610	S: FYM, W: feedlot, E/NW: CM, SW: alfalfa, N: house, out-buildings
	Fd2	Stanton	39.3	SE area	339	N/S: soy, W: SRA, E: feedlot, SW: FYM, SE: CM
	Fd3	Stanton	26.3	SE area	203	NE/SE: CM, E/N: FYM, S: soy, W: town of Pilger
	Fd4	Colfax	85	NE area	90	NE/W: soy, N: soy and alfalfa, E: CM, S: alfalfa
2022	Fd5	Boone	60.9	S area	46	S/N: CM, E: FYM
	Fd6	Stanton	60.7	SE area	90	S/E/W: CM; N: FYM
	Fd7	Stanton	30.9	SE area	137	S: pasture/prairie, E/W: CM, N: soy
	Fd8	Cuming	8.7	SE area	90	N/W/E: creek/wooded area, S: CM/part wooded area

^a^FYM = first-year maize, CM = continuous maize, SRA = State Recreation Area, soy = soybean, N = north, S = south, E = east, W = west.

### Field designs/data collection

Each field was an experimental unit or replicate. Four commercial first-year maize fields were included in the study each year during 2021 (Fd1-Fd4) and 2022 (F5-Fd8) to monitor WCR adult activity patterns (Table 1). To evaluate corn rootworm root injury and adult emergence in first-year maize, a hybrid with Lepidoptera-active traits but without rootworm-active traits (non-RW Bt) was planted in a subset of fields at 79,074 plants per ha (32,000 plants/A). Seed was treated with clothianidin at 0.5 mg/seed. A 4-row (3.1 m) x ca. 61.5 m length strip was planted without soil insecticide in first-year maize Fd2-Fd4 during 2021, and fields Fd5-Fd7 during 2022. A non-RW Bt strip without soil insecticide was also placed in second-year maize fields Fd2-Fd4 in 2022 to compare root injury and adult emergence in first-year and second-year maize. In both 2021 and 2022, all strips were planted within a similar May 5–15 window. Maize was managed using commercial practices appropriate for the region with farmers applying fertilizer and herbicides to strips as part of applications to the entire field each strip was placed in. Plots were kept weed-free.

#### Adult activity—first-year maize.

Pherocon AM unbaited sticky traps (Trécé Inc., Adair, OK) were placed at ear height in first-year maize to measure adult WCR activity in the primary feeding zone within fields. Eight traps were placed at least 30 rows into each field >30 m apart to provide spatially independent samples [42] in the area where non-RW Bt strips would be planted in second-year maize. Traps were placed after initial adult WCR activity was observed and were replaced on a weekly basis for nine collection periods in fields Fd1-Fd3 (13 July-15 September 2021), nine collection periods in Fd4 (6 July-7 September 2021) and Fd5 (13 July-15 September 2022), and six collection periods (14 July-25 August 2022) in fields Fd6-Fd8. Male and female WCR adults were counted per trap. The number of females exhibiting the visible presence or absence of egg development in a swollen abdomen was also recorded. No attempt was made to use published rating scales [43] to further classify egg development. The nine collection-period datasets covered most of the WCR adult activity period through maize dry-down. Adult collections in fields Fd6-Fd8 were terminated in 2022 prior to the end of the WCR activity period because the fields were harvested as high moisture maize or silage. The predominant maize growth stage [44] was recorded per field during each sample week.

#### Adult emergence.

Individual plant cages were placed over four total plants in one of the center two rows of each non-RW Bt strip. Each cage was ca.12 m apart. Emergence cage design allowed the caged plant to remain intact and grow up through the center of the cage [45]. Emerging WCR adults were collected in a glass jar that included an inverted paper Konie cup with tip cut off (Konie Cups International Inc., Miami, FL). Jars were changed weekly and the total number of WCR in each jar was counted.

#### Root injury.

During late July each year, individual plants were dug from the plant row that contained single-plant emergence cages to measure root injury. In total, 10 plants were randomly selected at ca. 4m intervals from each non-RW Bt strip. Roots were washed and injury rated using the 0–3 node injury scale (NIS [46]). WCR egg density levels and associated NIS ratings are highly correlated [47] so the associated general larval pressure present can be inferred from NIS ratings. NIS ratings were used as an indirect measure of larval pressure or density in this study.

### WCR single plant bioassays

#### WCR populations.

Adult WCR were annually collected during August 2021 and 2022 from most first-year maize fields included in the on-farm study. A minimum of 50 gravid females (usually >150) were collected from each field near the location where the on-farm strips would be placed the following year to obtain a subset of the natural variation present. Adults were not collected from fields Fd1 and Fd3 as densities were too low to meet the minimum criteria listed above. In 2021, adults were also collected from some adjacent or nearby continuous maize fields to gauge the susceptibility level of populations to Bt traits commonly planted in the landscape. Field-collected adult WCR were transported to the Department of Entomology at the University of Nebraska-Lincoln and maintained by population in 28 cm^3^ plexiglass cages under laboratory conditions during the summer and fall of each year. About 10,000 eggs were obtained per population each year. The procedural steps used to maintain adults, collect eggs, and the temperature regimens used to facilitate egg diapause and post-diapause development are described in Wangila et al. [18] and Reinders et al. [25].

Diapausing WCR colonies reared and maintained at the USDA-ARS North Central Agricultural Research Laboratory in Brookings, South Dakota, were used as lab control populations. Each control population was collected prior to the initial commercialization of Bt proteins in 2003 and was continuously reared without the addition of wild-type genes, preserving susceptibility to rootworm-active transgenic maize. Control populations originated from Butler County, Nebraska (1999, used in 2022 bioassays), and from York County, Nebraska (1996, used in 2023 bioassays).

#### Bioassay procedure.

Neonate progeny of the F_1_ generation from each population were used in bioassays as described by Gassmann et al. [16] and adapted by Wangila et al. [18] and Reinders et al. [25]. This standardized technique is used to detect shifts in WCR susceptibility to Bt proteins [16,19,23,24]. Bioassays were conducted during the spring to summer of the year following beetle collection after termination of obligatory egg diapause (e.g., 2023 bioassays conducted with progeny of 2022 field collections). Two sets of bioassays were conducted simultaneously with hybrids of different genetic backgrounds. The first set included three maize hybrids without seed treatments: single-protein Cry3Bb1, the Cry3Bb1 + Gpp34Ab1/Tpp35Ab1 pyramid, or no rootworm-Bt traits. The second set included two maize hybrids without seed treatments expressing Gpp34Ab1/Tpp35Ab1 or no rootworm-Bt traits. The same hybrids were used for all bioassays conducted during 2022–2023. Twelve plants of each hybrid were grown in individual 1L plastic pots (Johnson Paper & Supply Co., Minneapolis, MN) until the V4-V5 growth stage [44] to assay each WCR population. Twelve randomly selected F_1_ neonate larvae (≤24h after eclosion) were then placed on the roots of each individual plant and pots were held at 24°C with a 14:10 (L:D) photoperiod for 17 days. Each plant and surrounding soil was then placed in a separate Berlese funnel (40 W, 120 V lightbulbs) for 4 days to extract larval survivors. Seed was provided by Bayer CropScience (Cry3Bb1, Cry3Bb1 + Gpp34Ab1/Tpp35Ab1, no rootworm trait near isoline) and Corteva Agriscience (Gpp34Ab1/Tpp35Ab1, no rootworm trait near isoline) for use in bioassays.

### Data analysis

All data were analyzed using SAS 9.4 software [48]. Statistical significance was reported at α = 0.05 for all analyses.

#### Adult captures on sticky traps.

A general linear mixed model with the LSmeans option in SAS was used to determine if mean male and female counts on sticky traps were significantly different during each collection period. A separate analysis was conducted with the six collection-period (three replicates: Fd6-Fd8) and nine collection-period (four replicates: Fd1-Fd3, Fd5) datasets to group fields with similar WCR phenology and field sampling duration so each field within each analysis could be considered a replicate. This enabled general inferences to be made about WCR activity in the study area. Collection-period dates which started at first observance of WCR adults and collection-period duration were uniform across fields in each analysis. Fd4 was excluded from the nine collection-period analyses and included as a separate case history because initial beetle collection was earlier than other fields in the study and the farmer cooperator applied bifenthrin (rate: 365 ml/ha) on 4 August 2021 after collection period 5. Preliminary analysis of the nine collection-period dataset indicated overdispersion was present when a Poisson distribution was used which was remedied by using the negative binomial distribution and a random effect associated with field. The six collection-period data fit a Poisson distribution with random effects field and trap within field. Fixed effects were beetle sex, time, and time*beetle sex for each analysis. Simple effect contrasts of time*sex LSmeans were used to compare means within sex among periods. Sidaks multiplicity adjustment was used to control for experiment-wise error rate.

#### Adult emergence.

A Poisson hurdle model [49,50] was fit to the 2021 and 2022 adult emergence data from fields Fd2-Fd4 (three replicates per year) because standard Poisson or negative binomial general linear mixed models did not sufficiently account for the many zeros in the datasets (Table 2). Not accounting for the inflated frequency of zeros can disrupt mean estimation and comparisons between group means [49]. Count data was fitted using SAS PROC NLMIXED and year was included as a fixed effect. The model estimated the probability of zero adult emergence for 2021 and 2022 data plus compared the difference between years. In addition, the model estimated mean emergence per year when adult emergence did occur plus comparison of mean emergence in 2022 and 2021. Significant differences were determined with estimate statements within NLMIXED.

**Table 2:** Mean proportion western corn rootworm females with egg development per sampling period collected on unbaited Pherocon AM sticky traps.

Fields	Collection Period	Mean Proportion Female ± SE With Egg Development
Fd1-Fd3, Fd5	1	0.00
	2	0.53 ± 0.12
	3	0.76 ± 0.11
	4	0.92 ± 0.08
	5	0.86 ± 0.10
	6	1.00
	7	1.00
	8	1.00
	9	1.00
Fd6-Fd8	1	0.00
	2	0.99 ± 0.01
	3	1.00
	4	1.00
	5	1.00
	6	1.00
Fd4	1	0.00
	2	17.6 ± 0.02
	3	0.75 ± 0.04
	4	0.95 ± 0.01
	5	0.96 ± 0.01
	6	1,00
	7	1.00

Females were collected weekly in fields 1−3, and 5 from 13 July-15 Sept, fields 6−8 from 14 July-26 Aug, and field 4 from 13 July-1 Sept. No females were collected from field 4 during collection periods 8 and 9. Means were calculated from eight traps per field and four fields in the nine collection-period dataset, three fields in the six collection-period dataset. In field 4, means were calculated per period from females collected from independent trapping sites within the field. Female sample sizes per collection period are provided in S1 Table.

#### Root injury.

Node injury scores (root injury ratings) recorded from plants dug from each plot followed a continuous distribution within the restricted interval of 0–3 [46]. Root injury scores were transformed prior to analysis by dividing each score by 3 to calculate proportional root injury (0–1 scale). If the value was 0 it was set to 0.001 and any value equal to 1 was set to 0.999. A generalized linear mixed model with a beta distribution was used to compare mean 2021 versus 2022 transformed data from fields Fd2-Fd4 (three replicates per year). Year was a fixed factor, and field was included as a random effect in the model. The type III test of fixed effects was used to evaluate the significance of the fixed factor year.

#### Bioassay corrected survival.

Bioassay proportional survival was calculated on a per plant basis by dividing the number of larval survivors by the number of larvae infested per plant. Corrected survival on the Bt pyramid hybrid and each single Bt protein was calculated as survival on each Bt bioassay plant divided by mean survival on the non-RW Bt hybrid for each population [51]. A linear mixed model (implemented using PROC GLIMMIX) following a normal distribution with unequal variances between populations was used to evaluate corrected survival [24,52] within each Bt hybrid for each year assays were conducted (2022: Fd2, Fd4, 7 continuous maize populations, 1 lab control; 2023: Fd5-Fd8, 1 lab control). WCR population was included in the model as a fixed factor. Heterogenous variance between populations was allowed to control for nonconstant variance by specifying GROUP = Population in the random statement. The DIFFS option was used to identify significant differences in corrected survival among WCR populations within each Bt hybrid.

## Results

### Adult captures on sticky traps

**Fig 2:**
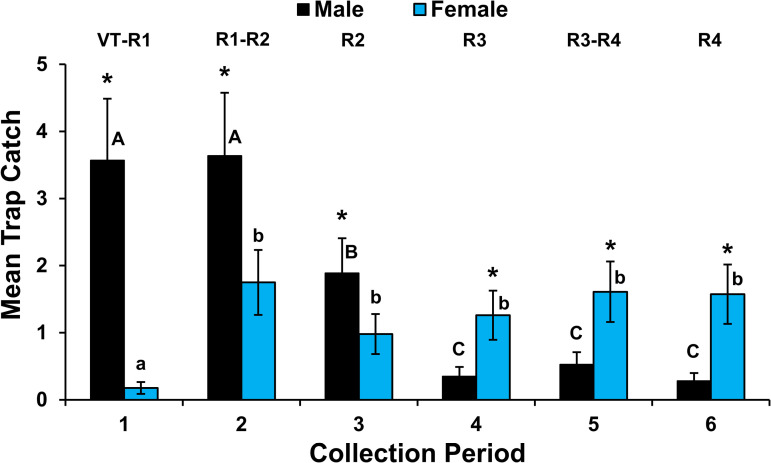
Mean ( **±SE) male and female western corn rootworm adults collected from first-year maize fields Fd6-Fd8 on Pherocon AM unbaited sticky traps placed at ear height per collection period.** Eight traps were placed >30 m apart per field and replaced weekly during six collection periods (13 July-26 August 2022) after initiation of adult WCR activity. An asterisk indicates a significant difference in mean male to female trap catch within collection period (general linear mixed model, *p < *0.05; LSMEANS option). Means with the same upper-case letter (males) or lower-case letter (females) are not significantly different (simple effect contrasts of time*sex LSmeans, *p* > 0.05). Sidaks multiplicity adjustment was used to control for experiment-wise error rate. Maize growth stages listed per collection period [44].

**Fig 3:**
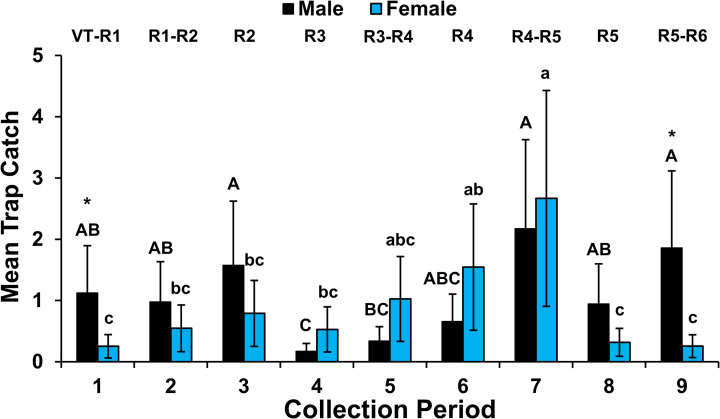
Mean ( **±SE) male and female western corn rootworm adults collected from first-year maize fields Fd1-Fd3, Fd5 on Pherocon AM unbaited sticky traps placed at ear height per collection period.** Eight traps were placed >30 m apart per field and replaced weekly during nine collection periods (13 July-15 September, Fd1-Fd3 2021; Fd5 2022) after initiation of adult WCR activity. An asterisk indicates a significant difference in mean male to female trap catch within collection period (general linear mixed model, *p < *0.05; LSMEANS option). Means with the same upper-case letter (males) or lower-case letter (females) are not significantly different (simple effect contrasts of time*sex LSmeans, *p* > 0.05). Sidaks multiplicity adjustment was used to control for experiment-wise error rate. Maize growth stages listed per collection period [44].

In the six collection-period dataset, the adult WCR male to female count comparisons were significantly affected by the fixed effects time (*F*_5, 253 _= 18.74, *p* < 0.0001) and time*beetle sex interaction (*F*_5, 253 _= 22.66, *p* < 0.0001). Mean male trap catch was significantly greater than mean female trap catch during the first three collection periods (Fig 2). This pattern was reversed during collection periods 4–6 as mean female trap catch became significantly greater than mean male trap catch (Fig 2). The fixed effects time and time*beetle sex interaction also significantly affected mean male to female count comparisons in the nine collection-period dataset (time: *F*_8, 555_ = 8.70, *p* < 0.0001; time*beetle sex interaction: *F*_8, 555 _= 6.67, *p* < 0.0001). A similar trend was exhibited in the nine collection-period dataset during the first six collection periods as observed in the six collection-period dataset, but the only significant difference occurred during the first period when mean male trap catch was greater than mean female trap catch (Fig 3). Mean male trap catch was also significantly greater than mean female trap catch in collection period 9 (Fig 3).

Simple effect contrasts within sex (S1 Table) in the six collection-period dataset indicated female trap catch was significantly lower in collection period 1 compared to other periods (Fig 2). Female trap catch was not significantly different among collection periods 2–6 (S1 Table, Fig 2). Male trap catch was significantly greatest during periods 1 and 2 with collections during periods 1–3 significantly greater than periods 4–6 (Fig 2).

In the nine collection-period dataset, simple effects contrasts within sex (S2 Table, Fig 3) indicated no significant difference in female trap catch among dates except for a spike in trap catch during periods 6 and 7 which was significantly greater than trap catches in periods 1, 8 and 9 (Fig 3). Male trap catch was significantly greater in collection periods 1–3 and 7–9 than period 4 (Fig 3). Male trap catch in period 5 was also significantly lower than trap catch in periods 7 and 9 (Fig 3).

**Fig 4:**
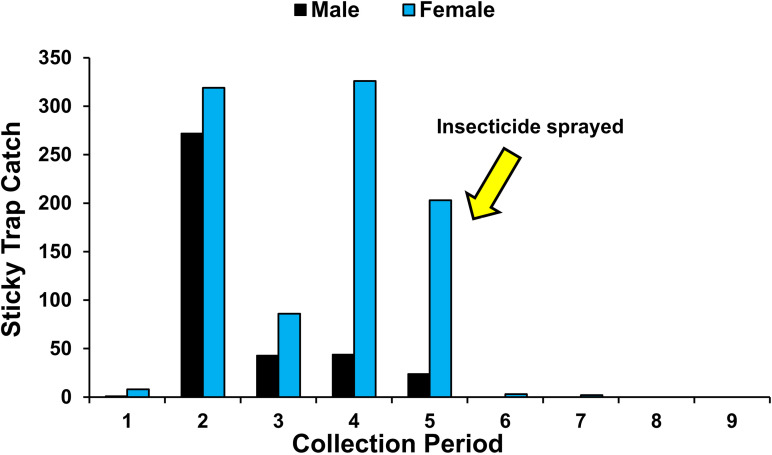
Total male and female western corn rootworm adults collected from first-year maize field Fd4 on Pherocon AM unbaited sticky traps placed at ear height per collection period. Eight traps were placed >30 m apart per field and replaced weekly during nine collection periods (6 July-7 September 2021) after initiation of adult WCR activity. Bifenthrin (rate: 365 ml/ha) was applied 4 August 2021 just after week five collection period.

Adult collections on sticky traps in Fd4 were very low in collection period 1 with a large increase in males and females collected during period 2 (Fig 4). Densities of each sex collected declined during week 3 but female collections rebounded to high levels during periods 4 and 5. Adult collections dropped to near zero or zero after insecticide was applied following collection period 5 (Fig 4).

Ovarian development of females collected on sticky traps followed a similar pattern in the first-year maize fields sampled. During week one when few females were collected (Figs 2–4, S3, S4 Tables) no egg development was observed (Table 2). In the following weeks, the proportion of females with visible egg development rapidly increased in collections until reaching 100% during mid to later collection periods (Table 2).

### Adult emergence

Seasonal adult emergence was very low in first-year maize (Table 3). Hurdle model results indicate that the probability of no adult emergence occurring in 2021 was significantly greater than the probability of no adult emergence in 2022 (Table 4). In addition, when emergence did occur, the mean number of adults collected in cages was significantly greater in 2022 than 2021 (Table 4).

**Table 3:** Summary of western corn rootworm emergence, sticky trap collection, and root injury ratings from each first-year and second-year maize field, 2021-2022.

Year	Field	Field Classification^a^	Total Adult Emergence^b^	Total Adults Caught On Sticky Traps^c^	Mean NIS ± SE^d^
2021	Fd1	FYM	0	32	0.00
	Fd2	FYM	7	87	0.02 ± 0.01
	Fd3	FYM	0	142	0.00
	Fd4	FYM	14	1334	0.02 ± 0.01
2022	Fd5	FYM	0	1025	0.02 ± 0,01
	Fd6	FYM	0	263	0.06 ± 0.01
	Fd7	FYM	0	97	0.05 ± 0.01
	Fd8	FYM	0	143	0.01 ± 0.01
2022	Fd2	SYM	83	NA	0.11 ± 0.07
	Fd3	SYM	5	NA	0.14 ± 0.03
	Fd4	SYM	90	NA	2.90 ± 0.06

^a^FYM=first-year maize, SYM=second-year maize

^b^total seasonal count from four single-plant emergence cages

^c^total seasonal count from eight unbaited Pherocon AM sticky traps

^d^NIS = node injury score, mean from sample of 10 roots, SE = standard error.

**Table 4:** Results of Poisson hurdle model analysis of western corn rootworm adult emergence from first-year and second-year maize fields Fd2-Fd4 in 2021 and 2022.

Parameter	Estimate ± SE	DF	t Value	Pr > t
2021: Probability of no emergence	0.8796 ± 0.031	192	28.09	<0.0001
2022: Probability of no emergence	0.6190 ± 0.052	192	11.68	<0.0001
2022 to 2021: no emergence comparison	0.2606 ± 0.061	192	4.23	<0.0001
2021: Emergence estimate	1.0502 ± 0.347	192	3.02	0.0029
2022 Emergence estimate	5.5406 ± 0.419	192	13.20	<0.0001
2022 to 2021: Emergence comparison	5.2760 ± 1.791	192	2.95	0.0036

^a^SE = standard error

### Root injury

Root injury was also very low in first-year maize (Table 3). The analysis of transformed injury scores from fields Fd2-Fd4 indicated significantly greater mean injury was recorded in 2022 than in 2021 (*F*_1,56 _= 20.97, *p* < 0.0001). Root injury score LSmeans were 0.13 ± 0.06 in 2021 and 0.44 ± 0.11 in 2022.

### Bioassay corrected survival

Significant variation in corrected survival occurred among populations in single trait Cry3Bb1, Gpp34Ab1/Tpp35Ab1 and the Cry3Bb1 + Gpp34Ab1/Tpp35Ab1 pyramid bioassays conducted during 2022 and 2023 (Cry3Bb1: 2022: *F*_9,34.14 _= 60.16, *p* < 0.0001; 2023: *F*_4,16.82 _= 40.2, *p* < 0.0001; Gpp34Ab1/Tpp35Ab1: 2022: *F*_9,37.34_ = 29.36, *p* < 0.0001; 2023: *F*_4,17.32_ = 15.00, *p* < 0.0001; pyramid: 2022: *F*_9, 34.12 _= 60.58, *p* < 0001; 2023: *F*_4,14.83_ = 47.77, *p* < 0.0001; Tables 5, 6). Populations bioassayed from the two first-year maize fields in 2022 (Fd2, Fd4) were highly resistant to both Cry3Bb1 (corrected survival ≥ 0.80) and Gpp34Ab1/Tpp35Ab1 (corrected survival ≥ 0.5) while 2023 bioassays revealed only two of four first-year maize populations were highly resistant to Cry3Bb1 (corrected survival: Fd6:1.0, Fd5: 0.60) and one to Gpp34Ab1/Tpp35Ab1 (Fd6: corrected survival = 0.61). Three populations bioassayed in 2023 (Fd5, Fd7, Fd8) exhibited significantly lower corrected survival than Fd6 to Cry3Bb1 (corrected survival range: 0.31–0.60) and Gpp34Ab1/Tpp35Ab1 (corrected survival range: 0.17–0.37). Pyramid bioassay results tended to align with single Bt trait bioassay results (Tables 5,6). Lab control corrected survival was very low each year and was significantly different in each trait bioassay than populations collected from first-year or continuous maize (Tables 5, 6).

**Table 5:** Corrected survival of western corn rootworm populations on Bt maize in bioassays conducted during 2022.

County	Field/ Population	Field Classification^a^	Cry3Bb1 Corrected Survival ± SE^b^	Cry3Bb1 + Gpp34/Tpp35Ab1 Corrected Survival ± SE^b^	Gpp34/Tpp35Ab1 Corrected Survival ± SE^b^
Stanton	Fd2	FYM	0.961 ± 0.09a	0.794 ± 0.09a	0.566 ± 0.05bc
Colfax	Fd4	FYM	0.800 ± 0.07ab	0.491 ± 0.07c	0.500 ± 0.07cde
Stanton	NW Fd2	CM	0.904 ± 0.17ab	0.962 ± 0.17a	0.638 ± 0.06abc
Boone	E Fd5	CM	0.800 ± 0.14ab	0.675 ± 0.14abc	0.344 ± 0.05e
Boone	S Fd5	CM	0.903 ± 0.11ab	0.403 ± 0.11c	0.561 ± 0.06bc
Stanton	SE Fd2	CM	0.660 ± 0.07b	0.564 ± 0.07bc	0.391 ± 0.04de
Colfax	SW Fd4	CM	0.825 ± 0.08ab	0.660 ± 0.08abc	0.744 ± 0.09ab
Colfax	E Fd4	CM	0.880 ± 0.09ab	0.587 ± 0.09abc	0.552 ± 0.08bcd
Stanton	NE Fd3	CM	0.866 ± 0.11ab	0.794 ± 0.11ab	0.782 ± 0.07a
	Lab Control	N/A	0.040 ± 0.02c	0.013 ± 0.02d	0.053 ± 0.02f

^a^Field classifications: FYM = first-year maize; CM = continuous maize; N/A = not applicable

^b^Within columns, corrected survival values followed by the same lowercase letter are not significantly different (Linear mixed model, normal distribution, DIFFS option in SAS: *p* > 0.05), SE = standard error.

$\begin{array}{c} \;Corrected\;survival=1-\frac{survival\;on\;isoline-survival\;\;on\;\;Bt}{survival\;on\;isoline} \end{array}$

**Table 6:** Corrected survival of western corn rootworm populations on Bt maize in bioassays conducted during 2023.

County	Field/Population^a^	Cry3Bb1 Corrected Survival ± SE^b^	Cry3Bb1 + Gpp34/Tpp35Ab1 Corrected Survival ± SE^b^	Gpp34/Tpp35Ab1 Corrected Survival ± SE^b^
Boone	Fd5	0.600 ± 0.08b	0.400 ± 0.08b	0.196 ± 0.06bc
Stanton	Fd6	1.000 ± 0.13a	0.828 ± 0.13a	0.614 ± 0.09a
Stanton	Fd7	0.484 ± 0.05b	0.355 ± 0.05b	0.367 ± 0.07b
Cuming	Fd8	0.310 ± 0.04c	0.207 ± 0.04c	0.167 ± 0.05c
	Lab control	0.014 ± 0.01d	0.000 ± 0.00d	0.029 ± 0.02d

^a^All fields were first-year maize

^b^Within columns, corrected survival values followed by the same lowercase letter are not significantly different (Linear mixed model, normal distribution, DIFFS option in SAS: *p* > 0.05), SE = standard error. $\begin{array}{c} Corrected\;survival=1-\frac{survival\;on\;isoline-survival\;on\;Bt}{survival\;\;on\;\;isoline} \end{array}$

## Discussion

Variability in temporal and spatial relationships greatly impacts movement and population dynamics of the WCR [6,39]. This was clearly observed in first-year maize during this study. Zero to very low adult emergence from within first-year maize indicates that initial trap catches in first year maize came primarily from immigrants arriving from source fields or from rare individuals emerging within fields. As adults accumulated in first year maize, trap catch would have been the result of intra-field movement and additional immigration. Adult WCR activity patterns in the first six periods of both six collection-period and nine collection-period datasets are consistent with previous studies (hand collections [53]; yellow sticky traps [40]; sweep net samples [54] that have reported a male bias during early sampling periods followed by a shift to more females than males. Early male bias could be related to initiation of male emergence before females [4,55] and greater male movement prior to mating than females [4,56,57]. In source continuous maize fields during the early emergence period, male reproductive potential may be much greater than the number of available virgin females, leading to intraspecific competition between males [58]. This could contribute to ranging behavior of males outside of the natal field.

In general, WCR beetle collections at ear zone height from unbaited sticky traps are more skewed toward males than hand-aspirated collections [40,59] so a significant reduction in mean male catch in this study during collection periods 4–6 suggests either a male behavioral change (less activity around ear zone) or density reduction after the R2 corn growth stage. Whole plant counts or aspirated adult collections were not included in this study so it is not clear if a density reduction of the male population did occur.

Most females probably mated in source fields then moved to first-year maize as mating usually takes place near the emergence site within hours of emergence [4,56,60,61]. Initial female movement into first-year maize may have been the result of short-range dispersal and possibly longer-range migration as both occur during the preovipositional period after mating [4,37,39]. If any unmated females were present in first-year maize they were probably quickly mated because of the significant male activity present. Most females only mate once [4,61] so the high proportion of females with visible egg development by sampling period 3 may have triggered some males to move out of the field in search of unmated females.

Many females in a typical maize field are gravid (fully developed eggs) during the R2-R4 growth stages [40,53] and may remain in maize longer than males to oviposit. In this study, gravid females may have spent time at ground level exhibiting ovipositional behaviors [62,63] and periodically moving vertically to the ear zone to feed on ear tips. This may have contributed to relatively stable mean female trap catch densities during maize growth stages R1-R4.

In the nine collection-period dataset, the significant increase in mean male and female trap-catch during collection period 7 suggests that both sexes were exhibiting greater intra- and interfield movement in search of suitable food sources during R4-5 maize growth stages. Increased flight behavior may have made the yellow sticky traps more apparent and attractive leading to higher mean trap catches of mobile beetles. Advanced crop maturity leading to unfavorable adult host conditions has been previously linked to increased adult movement within and among maize fields [55,64,65] and increased utilization of pollinating weeds outside of maize as nutritional sources [66–68]. Elliott et al. [69] reported significant reduction in survival of adults when held on maize growth stages R5-R6 [44] and documented young ear tissue and/or pollen feeding was needed to initiate and maintain female WCR egg production. The rapid significant reduction in mean female trap catch during collection periods 8 and 9 suggests movement occurred out of the field during maize growth stages R4-R5 because maize became a poor adult host.

It is interesting to note that mean male trap catch did not significantly decline during collection periods 7–9 in the nine collection-period dataset and was significantly greater than mean female trap catch during week 9 (Fig 3). However, variation in male and female trends was great among fields late in the season (S3 Table, Fig 3: large SE in periods 7, 9). Trap catch in Fd1-Fd3 was skewed toward males in late collection periods while trap catch in Fd5 exhibited the consistent female bias in first-year maize late in the growing season reported by Godfrey and Turpin [40] who also used unbaited Pherocon AM traps. The greater male trap catch observed in Fd1-Fd3 during collection periods 7–9 than periods 4 and 5 in this study (S3 Table) suggests that male movement increased in the landscape late in the growing season and indicates active males were present in some maize fields even though maize became a poor nutritional source.

In the transgenic era, the frequency and level of WCR Bt resistance in source fields may have contributed to the variability in late-season male/female dynamics among first-year maize fields observed in this study. In Bt susceptible populations or when low levels of Bt resistance are present, dietary exposure of WCR larvae to Bt maize can slow larval developmental rates and shift peak emergence of males and females to later in the season [70–75] which will potentially produce younger males and some teneral females in September [56]. Males mate multiple times but mainly when younger in age [76] so late season emerging males may be exhibiting ranging behavior to locate females emerging in the mosaic of Bt maize fields present in the landscape. This is supported by mean male trap catches during early and late season not being significantly different (Fig 3). The delay in WCR larval development decreases as Bt resistance levels increase until emergence patterns are similar in non-Bt refuge maize and Bt maize when high levels of Bt resistance are present [25,41,52]. Additional study would be needed to test the hypothesis that the mosaic of WCR Bt resistance levels and associated adult emergence patterns in the landscape contributed to the late season results obtained in Fd1-Fd3 and Fd5.

Many factors such as distance to source fields, number of source fields near first-year maize, WCR densities in source populations, adult and host phenological interactions (discussed previously), or movement barriers (e.g., urban areas, stream/lake habitats with forest, feedlots, etc.) could all interact to impact WCR pattern and rate of immigration into first-year maize making it difficult to pinpoint key factors driving immigration. WCR adult densities were not measured in potential source fields but fields adjacent to Fd4 and Fd5 had high adult densities present (LJM personal observation). Fields Fd1-Fd3, Fd7, and Fd8 had one or more barriers to adult movement adjacent to fields (Table 1). Fields Fd5 and Fd6 had multiple adjacent potential source fields (Table 1). Beckler et al. [77] and Szalai et al. [78] reported that adjacent continuous maize fields were major sources of immigrants collected in first-year maize. In this study, a trend was evident that trap catches in first-year maize increased as distance from source fields decreased (i.e.,: Fd4, Fd5 versus Fd1, Fd2, Tables 1, 2). However, because WCR densities were not measured in source fields, the distance*adult density interaction could confound a comparison of distance*trap catch alone (especially in Fd4, Fd5 with known high densities). Tethered flight studies consistently have shown short range or appetitive WCR flights make up a large portion of total flights with longer-range migratory flights less common [37–39]. Therefore, appetitive flight could account for much of the local male and female activity observed within and among adjacent fields in this study [39].

Hurdle model analyses of fields Fd2-Fd4 indicating significantly greater mean root injury and increased adult emergence in second-year than first-year maize documented that immigrant WCR adults were ovipositing and colonizing first-year maize. This trend has been reported in previous field studies in which field densities increased in continuous maize over years after colonization of first-year maize [7,78]. Fields Fd2-Fd4 provide examples of the extreme variation in immigration and colonization that can occur in first-year maize which greatly impacted the level of injury recorded in second-year maize (Table 2). Even though the bifenthrin application to Fd4 appeared to provide excellent adult WCR control, the high level of root injury recorded in second-year maize (Table 2) indicates application timing was late and much oviposition occurred prior to the insecticide application (Fig 4).

This study provides empirical evidence documenting that resistant Bt alleles can be introduced when adult WCR colonize first-year maize. This supports published examples where introgression of resistant alleles into existing WCR populations was inferred to explain the presence of Bt resistance when little to no selection pressure had been observed (Cry3 resistance [25,79]; Gpp34Ab1/Tpp35Ab1 resistance [8,19]). In the system studied, WCR Bt resistance was present in all field populations sampled which suggests that levels of Bt resistance were pervasive in the landscape so the IRM benefit of within-field non-RW Bt refuge in Bt maize fields and the potential spatial refuge effect from the surrounding landscape appeared to be limited. As WCR Bt resistance spreads through the landscape, the frequency of Bt susceptible individuals is reduced so the functional IRM value of refuge will decline [11]. Models of IPM scenarios to mitigate Bt resistance suggest crop rotation is most effective as a WCR IRM tool right after initial resistance is detected when the surrounding landscape includes primarily susceptible individuals [35].

The 2022 Bt bioassay data indicated the high level of Bt resistance observed in first-year maize was not significantly different than Bt resistance present in many adjacent continuous maize fields (especially Cry3Bb1, Table 5). This was clearly observed in Fd4 and the adjacent continuous maize field directly east of Fd4 which had very similar corrected survival profiles (Table 5). This supports earlier discussion that suggested adjacent fields served as major sources of immigrants collected in first-year maize. Adult WCR collections were not made from adjacent fields in 2022, but the generally lower Bt resistance levels in 3 of 4 first-year maize fields in 2023 bioassays suggests that some source populations exhibited lower Bt resistance levels or contributed enough Bt susceptible immigrants to dilute the impact of highly Bt resistant immigrants on mean susceptibility level present. This dilution effect was inferred by Reinders et al. [25] where Cry3 resistance level in a field (field 6 in study) was lower than expected when continuous selection with Cry3 expressing hybrids for six consecutive years produced a relatively low WCR Cry3Bb1 corrected survival = 0.22. The result was attributed to immigration from large WCR populations of Bt susceptible adults from surrounding fields with no history of Cry3 hybrid use.

Current evidence clearly shows WCR Bt resistance evolves at the local level and is positively related to continuous use of the same Bt trait over multiple WCR generations [2,11,16,18,25]. This coupled with local WCR dispersal that impacts population dynamics and movement of resistant alleles, suggests farm/field level crop and IPM decisions made by farmers are important drivers of selection pressure and Bt resistance that occurs on their farms [2,11,25]. WCR density management is a key component of any WCR IPM or resistance mitigation strategy. When moderate-high levels of Bt resistance exist, root injury to Bt pyramids increases as WCR larval pressure (density) increases [8]. This study shows that a mosaic of WCR densities and Bt resistance levels can occur in first-year maize in a landscape that includes large areas of continuous maize with long-time use of rootworm Bt-trait hybrids. A mosaic of WCR susceptibility to Bt traits and insecticides has also been reported from continuous maize in the same region of Nebraska [8,23,24,80]. Because of this, scouting of first-year maize is recommended to match appropriate WCR management approaches to relative risk of injury in second-year maize (i.e., Fd1-Fd3 low-risk; Fd4, Fd5 high-risk). More intensive management would be needed in high-risk fields to reduce WCR density and potential injury to acceptable levels in second-year maize (see [2,8,81] for overview of management options). Scouting and management adjustments should annually be repeated in the following years if a continuous maize agronomic practice is resumed.

In summary, this study provides insight into WCR immigration and subsequent activity in first-year maize in the transgenic era, plus associated movement of Bt resistant alleles by WCR immigrants from the surrounding landscape. A complex set of factors contributes to variability in the level of WCR immigration and level of Bt resistance recorded among maize fields [2,11,25,39]. Maize and WCR phenological interactions plus gender-related behaviors appeared to be key determinants of WCR activity in first-year maize. Increased WCR male activity was documented in some first-year maize fields late in the season which warrants further study to understand underlying mechanisms involved and the potential impact of late-season matings on population dynamics and Bt resistance levels. Both short and long-range WCR movement could have contributed to the immigrating populations in first-year maize [39] but data presented in this paper support adjacent continuous maize fields as major contributors of individuals colonizing first-year maize. Results from this study support previous reports that documented crop rotation to a non-WCR host can eliminate the resident population in a field for at least one generation [2,3,39]. However, when specific factors are present, recolonization of high densities of Bt resistant WCR from source fields can quickly occur in first-year maize. Scouting of first-year maize is recommended to determine appropriate WCR management approaches to reduce relative risk of injury in second-year maize. In areas where WCR resistance to Bt traits is common, farmers can manage WCR density to mitigate the negative impacts of Bt resistance and reduce WCR injury by implementing different tactics as needed within an IPM framework [2,8]. Long-term field/farm-level WCR strategies over multiple seasons including field-level scouting plus crop and other tactic rotations should be considered.

## Supporting information

S1 Table Simple Effect Comparisons of time*sex Least Squares Means by sex (f = female, m = male) for western corn rootworm adults collected on Pherocon AM unbaited sticky traps, six collection-period dataset; Sidak adjustment for multiple comparisons. (DOCX)

S2 Table Simple Effect Comparisons of time*sex Least Squares Means by sex (f = female, m = male) for western corn rootworm adults collected weekly on Pherocon AM unbaited sticky traps, nine collection-period dataset; Sidak adjustment for multiple comparisons. (DOCX)

S3 Table Western corn rootworm adults caught on Pherocon AM unbaited sticky traps by field, collection period, trap; and sex; nine collection-period dataset, 13 July – 15 September 2021 (Fd1-Fd3), 2022 (Fd5). (DOCX)

S4 Table Western corn rootworm adults caught on Pherocon AM unbaited sticky traps by field, collection period, trap, and sex; six collection-period dataset, 13 July – 25 August 2022 (Fd6-Fd8). (DOCX)
