# Supplementary material for: Western corn rootworm adult activity and immigrant resistance to Bt traits in first-year maize
Source: PLoS One. 2025 Jun 13;20(6):e0325388. doi: 10.1371/journal.pone.0325388 (PMC12165417; doi:10.1371/journal.pone.0325388)
Supplement: S1 Table — (DOCX) [file pone.0325388.s001.docx]

| **S1 Table. Simple Effect Comparisons of time*sex Least Squares Means by sex (f=female, m=male) for western corn rootworm adults collected on Pherocon AM unbaited sticky traps, six collection-period dataset; Sidak adjustment for multiple comparisons** | | | | | | | | | | | | | |
| --- | --- | --- | --- | --- | --- | --- | --- | --- | --- | --- | --- | --- | --- |
| **Simple Effect Level** | **Period** | **_Period** | **Estimate** | **Standard Error** | **DF** | **t Value** | **Pr > \|t\|** | **Adj P** | **Alpha** | **Lower** | **Upper** | **Adj Lower** | **Adj Upper** |
| **sex f** | **1** | **2** | -2.3022 | 0.4690 | 253 | -4.91 | <.0001 | <.0001 | 0.05 | -3.2258 | -1.3787 | -3.6884 | -0.9161 |
| **sex f** | **1** | **3** | -1.7224 | 0.4854 | 253 | -3.55 | 0.0005 | 0.0069 | 0.05 | -2.6784 | -0.7664 | -3.1572 | -0.2876 |
| **sex f** | **1** | **4** | -1.9737 | 0.4772 | 253 | -4.14 | <.0001 | 0.0007 | 0.05 | -2.9135 | -1.0339 | -3.3841 | -0.5633 |
| **sex f** | **1** | **5** | -2.2189 | 0.4708 | 253 | -4.71 | <.0001 | <.0001 | 0.05 | -3.1461 | -1.2917 | -3.6105 | -0.8273 |
| **sex f** | **1** | **6** | -2.1969 | 0.4713 | 253 | -4.66 | <.0001 | <.0001 | 0.05 | -3.1251 | -1.2686 | -3.5900 | -0.8037 |
| **sex f** | **2** | **3** | 0.5798 | 0.2360 | 253 | 2.46 | 0.0147 | 0.1992 | 0.05 | 0.1150 | 1.0447 | -0.1179 | 1.2775 |
| **sex f** | **2** | **4** | 0.3285 | 0.2186 | 253 | 1.50 | 0.1341 | 0.8846 | 0.05 | -0.1019 | 0.7590 | -0.3175 | 0.9746 |
| **sex f** | **2** | **5** | 0.08337 | 0.2043 | 253 | 0.41 | 0.6836 | 1.0000 | 0.05 | -0.3190 | 0.4857 | -0.5205 | 0.6872 |
| **sex f** | **2** | **6** | 0.1054 | 0.2055 | 253 | 0.51 | 0.6085 | 1.0000 | 0.05 | -0.2993 | 0.5101 | -0.5020 | 0.7127 |
| **sex f** | **3** | **4** | -0.2513 | 0.2520 | 253 | -1.00 | 0.3196 | 0.9969 | 0.05 | -0.7475 | 0.2450 | -0.9960 | 0.4935 |
| **sex f** | **3** | **5** | -0.4964 | 0.2397 | 253 | -2.07 | 0.0394 | 0.4525 | 0.05 | -0.9685 | -0.02439 | -1.2049 | 0.2120 |
| **sex f** | **3** | **6** | -0.4744 | 0.2407 | 253 | -1.97 | 0.0498 | 0.5353 | 0.05 | -0.9485 | -0.00040 | -1.1859 | 0.2370 |
| **sex f** | **4** | **5** | -0.2452 | 0.2225 | 253 | -1.10 | 0.2716 | 0.9914 | 0.05 | -0.6834 | 0.1931 | -0.9029 | 0.4126 |
| **sex f** | **4** | **6** | -0.2232 | 0.2236 | 253 | -1.00 | 0.3192 | 0.9969 | 0.05 | -0.6635 | 0.2172 | -0.8841 | 0.4378 |
| **sex f** | **5** | **6** | 0.02201 | 0.2097 | 253 | 0.10 | 0.9165 | 1.0000 | 0.05 | -0.3909 | 0.4349 | -0.5977 | 0.6417 |
| **sex m** | **1** | **2** | -0.01943 | 0.1394 | 253 | -0.14 | 0.8892 | 1.0000 | 0.05 | -0.2939 | 0.2550 | -0.4313 | 0.3925 |
| **sex m** | **1** | **3** | 0.6360 | 0.1683 | 253 | 3.78 | 0.0002 | 0.0029 | 0.05 | 0.3045 | 0.9674 | 0.1385 | 1.1334 |
| **sex m** | **1** | **4** | 2.3221 | 0.3313 | 253 | 7.01 | <.0001 | <.0001 | 0.05 | 1.6696 | 2.9746 | 1.3428 | 3.3014 |
| **sex m** | **1** | **5** | 1.9170 | 0.2765 | 253 | 6.93 | <.0001 | <.0001 | 0.05 | 1.3724 | 2.4616 | 1.0996 | 2.7344 |
| **sex m** | **1** | **6** | 2.5455 | 0.3671 | 253 | 6.93 | <.0001 | <.0001 | 0.05 | 1.8224 | 3.2685 | 1.4603 | 3.6307 |
| **sex m** | **2** | **3** | 0.6554 | 0.1677 | 253 | 3.91 | 0.0001 | 0.0018 | 0.05 | 0.3251 | 0.9857 | 0.1596 | 1.1512 |
| **sex m** | **2** | **4** | 2.3415 | 0.3310 | 253 | 7.07 | <.0001 | <.0001 | 0.05 | 1.6896 | 2.9935 | 1.3631 | 3.3200 |
| **sex m** | **2** | **5** | 1.9364 | 0.2762 | 253 | 7.01 | <.0001 | <.0001 | 0.05 | 1.3925 | 2.4804 | 1.1200 | 2.7528 |
| **sex m** | **2** | **6** | 2.5649 | 0.3669 | 253 | 6.99 | <.0001 | <.0001 | 0.05 | 1.8424 | 3.2875 | 1.4805 | 3.6493 |
| **sex m** | **3** | **4** | 1.6861 | 0.3442 | 253 | 4.90 | <.0001 | <.0001 | 0.05 | 1.0082 | 2.3640 | 0.6687 | 2.7036 |
| **sex m** | **3** | **5** | 1.2810 | 0.2919 | 253 | 4.39 | <.0001 | 0.0003 | 0.05 | 0.7062 | 1.8558 | 0.4183 | 2.1437 |
| **sex m** | **3** | **6** | 1.9095 | 0.3788 | 253 | 5.04 | <.0001 | <.0001 | 0.05 | 1.1634 | 2.6556 | 0.7898 | 3.0292 |
| **sex m** | **4** | **5** | -0.4051 | 0.4082 | 253 | -0.99 | 0.3219 | 0.9971 | 0.05 | -1.2091 | 0.3988 | -1.6117 | 0.8015 |
| **sex m** | **4** | **6** | 0.2234 | 0.4743 | 253 | 0.47 | 0.6381 | 1.0000 | 0.05 | -0.7107 | 1.1575 | -1.1786 | 1.6253 |
| **sex m** | **5** | **6** | 0.6285 | 0.4378 | 253 | 1.44 | 0.1524 | 0.9162 | 0.05 | -0.2337 | 1.4907 | -0.6655 | 1.9225 |
