# Supplementary material for: Western corn rootworm adult activity and immigrant resistance to Bt traits in first-year maize
Source: PLoS One. 2025 Jun 13;20(6):e0325388. doi: 10.1371/journal.pone.0325388 (PMC12165417; doi:10.1371/journal.pone.0325388)
Supplement: S2 Table — (DOCX) [file pone.0325388.s002.docx]

| **S2 Table. Simple Effect Comparisons of time*sex Least Squares Means by sex (f=female, m=male) for western corn rootworm adults collected weekly on Pherocon AM unbaited sticky traps, nine collection-period dataset;**  **Sidak adjustment for multiple comparisons.** | | | | | | | | | | | | | |
| --- | --- | --- | --- | --- | --- | --- | --- | --- | --- | --- | --- | --- | --- |
| **Simple Effect Level** | **period** | **_period** | **Estimate** | **Standard Error** | **DF** | **t Value** | **Pr > \|t\|** | **Adj P** | **Alpha** | **Lower** | **Upper** | **Adj Lower** | **Adj Upper** |
| **sex f** | **1** | **2** | -0.7739 | 0.5358 | 555 | -1.44 | 0.1492 | 0.9970 | 0.05 | -1.8264 | 0.2786 | -2.4917 | 0.9440 |
| **sex f** | **1** | **3** | -1.1422 | 0.5175 | 555 | -2.21 | 0.0277 | 0.6365 | 0.05 | -2.1588 | -0.1257 | -2.8014 | 0.5169 |
| **sex f** | **1** | **4** | -0.7397 | 0.5364 | 555 | -1.38 | 0.1684 | 0.9987 | 0.05 | -1.7932 | 0.3138 | -2.4592 | 0.9798 |
| **sex f** | **1** | **5** | -1.4040 | 0.5087 | 555 | -2.76 | 0.0060 | 0.1939 | 0.05 | -2.4032 | -0.4048 | -3.0348 | 0.2268 |
| **sex f** | **1** | **6** | -1.8141 | 0.4979 | 555 | -3.64 | 0.0003 | 0.0105 | 0.05 | -2.7921 | -0.8360 | -3.4103 | -0.2178 |
| **sex f** | **1** | **7** | -2.3595 | 0.4899 | 555 | -4.82 | <.0001 | <.0001 | 0.05 | -3.3217 | -1.3973 | -3.9299 | -0.7890 |
| **sex f** | **1** | **8** | -0.2290 | 0.5614 | 555 | -0.41 | 0.6835 | 1.0000 | 0.05 | -1.3316 | 0.8737 | -2.0286 | 1.5707 |
| **sex f** | **1** | **9** | -0.01100 | 0.5784 | 555 | -0.02 | 0.9848 | 1.0000 | 0.05 | -1.1470 | 1.1250 | -1.8652 | 1.8432 |
| **sex f** | **2** | **3** | -0.3684 | 0.4231 | 555 | -0.87 | 0.3843 | 1.0000 | 0.05 | -1.1995 | 0.4627 | -1.7248 | 0.9881 |
| **sex f** | **2** | **4** | 0.03418 | 0.4467 | 555 | 0.08 | 0.9390 | 1.0000 | 0.05 | -0.8432 | 0.9116 | -1.3979 | 1.4662 |
| **sex f** | **2** | **5** | -0.6301 | 0.4119 | 555 | -1.53 | 0.1266 | 0.9924 | 0.05 | -1.4392 | 0.1789 | -1.9506 | 0.6904 |
| **sex f** | **2** | **6** | -1.0402 | 0.3974 | 555 | -2.62 | 0.0091 | 0.2803 | 0.05 | -1.8207 | -0.2597 | -2.3141 | 0.2337 |
| **sex f** | **2** | **7** | -1.5856 | 0.3894 | 555 | -4.07 | <.0001 | 0.0019 | 0.05 | -2.3505 | -0.8207 | -2.8341 | -0.3371 |
| **sex f** | **2** | **8** | 0.5449 | 0.4775 | 555 | 1.14 | 0.2543 | 1.0000 | 0.05 | -0.3930 | 1.4828 | -0.9859 | 2.0757 |
| **sex f** | **2** | **9** | 0.7629 | 0.4979 | 555 | 1.53 | 0.1261 | 0.9922 | 0.05 | -0.2152 | 1.7409 | -0.8334 | 2.3592 |
| **sex f** | **3** | **4** | 0.4026 | 0.4235 | 555 | 0.95 | 0.3422 | 1.0000 | 0.05 | -0.4293 | 1.2344 | -0.9552 | 1.7603 |
| **sex f** | **3** | **5** | -0.2617 | 0.3860 | 555 | -0.68 | 0.4979 | 1.0000 | 0.05 | -1.0199 | 0.4964 | -1.4991 | 0.9756 |
| **sex f** | **3** | **6** | -0.6718 | 0.3698 | 555 | -1.82 | 0.0698 | 0.9261 | 0.05 | -1.3982 | 0.05461 | -1.8574 | 0.5138 |
| **sex f** | **3** | **7** | -1.2172 | 0.3620 | 555 | -3.36 | 0.0008 | 0.0293 | 0.05 | -1.9282 | -0.5062 | -2.3776 | -0.05681 |
| **sex f** | **3** | **8** | 0.9133 | 0.4555 | 555 | 2.00 | 0.0455 | 0.8127 | 0.05 | 0.01852 | 1.8080 | -0.5471 | 2.3736 |
| **sex f** | **3** | **9** | 1.1312 | 0.4771 | 555 | 2.37 | 0.0181 | 0.4815 | 0.05 | 0.1941 | 2.0684 | -0.3984 | 2.6608 |
| **sex f** | **4** | **5** | -0.6643 | 0.4123 | 555 | -1.61 | 0.1077 | 0.9834 | 0.05 | -1.4741 | 0.1455 | -1.9860 | 0.6574 |
| **sex f** | **4** | **6** | -1.0744 | 0.3977 | 555 | -2.70 | 0.0071 | 0.2268 | 0.05 | -1.8556 | -0.2931 | -2.3494 | 0.2007 |
| **sex f** | **4** | **7** | -1.6198 | 0.3899 | 555 | -4.15 | <.0001 | 0.0014 | 0.05 | -2.3857 | -0.8539 | -2.8699 | -0.3697 |
| **sex f** | **4** | **8** | 0.5107 | 0.4780 | 555 | 1.07 | 0.2858 | 1.0000 | 0.05 | -0.4281 | 1.4496 | -1.0216 | 2.0430 |
| **sex f** | **4** | **9** | 0.7287 | 0.4984 | 555 | 1.46 | 0.1443 | 0.9963 | 0.05 | -0.2503 | 1.7076 | -0.8691 | 2.3265 |
| **sex f** | **5** | **6** | -0.4101 | 0.3570 | 555 | -1.15 | 0.2512 | 1.0000 | 0.05 | -1.1112 | 0.2911 | -1.5545 | 0.7343 |
| **sex f** | **5** | **7** | -0.9555 | 0.3485 | 555 | -2.74 | 0.0063 | 0.2037 | 0.05 | -1.6400 | -0.2709 | -2.0727 | 0.1618 |
| **sex f** | **5** | **8** | 1.1750 | 0.4452 | 555 | 2.64 | 0.0085 | 0.2655 | 0.05 | 0.3006 | 2.0494 | -0.2521 | 2.6021 |
| **sex f** | **5** | **9** | 1.3930 | 0.4672 | 555 | 2.98 | 0.0030 | 0.1023 | 0.05 | 0.4753 | 2.3107 | -0.1049 | 2.8908 |
| **sex f** | **6** | **7** | -0.5454 | 0.3315 | 555 | -1.65 | 0.1005 | 0.9779 | 0.05 | -1.1966 | 0.1058 | -1.6082 | 0.5174 |
| **sex f** | **6** | **8** | 1.5851 | 0.4319 | 555 | 3.67 | 0.0003 | 0.0095 | 0.05 | 0.7368 | 2.4334 | 0.2006 | 2.9696 |
| **sex f** | **6** | **9** | 1.8031 | 0.4548 | 555 | 3.96 | <.0001 | 0.0030 | 0.05 | 0.9097 | 2.6964 | 0.3449 | 3.2612 |
| **sex f** | **7** | **8** | 2.1305 | 0.4222 | 555 | 5.05 | <.0001 | <.0001 | 0.05 | 1.3013 | 2.9597 | 0.7771 | 3.4839 |
| **sex f** | **7** | **9** | 2.3485 | 0.4450 | 555 | 5.28 | <.0001 | <.0001 | 0.05 | 1.4744 | 3.2225 | 0.9219 | 3.7750 |
| **sex f** | **8** | **9** | 0.2180 | 0.5239 | 555 | 0.42 | 0.6775 | 1.0000 | 0.05 | -0.8111 | 1.2470 | -1.4615 | 1.8975 |
| **sex m** | **1** | **4** | 1.8789 | 0.4935 | 555 | 3.81 | 0.0002 | 0.0056 | 0.05 | 0.9096 | 2.8483 | 0.2968 | 3.4610 |
| **sex m** | **1** | **5** | 1.2005 | 0.4358 | 555 | 2.75 | 0.0061 | 0.1967 | 0.05 | 0.3445 | 2.0565 | -0.1966 | 2.5976 |
| **sex m** | **1** | **6** | 0.5372 | 0.4130 | 555 | 1.30 | 0.1939 | 0.9996 | 0.05 | -0.2741 | 1.3486 | -0.7869 | 1.8614 |
| **sex m** | **1** | **7** | -0.6598 | 0.3896 | 555 | -1.69 | 0.0909 | 0.9676 | 0.05 | -1.4250 | 0.1054 | -1.9087 | 0.5891 |
| **sex m** | **1** | **8** | 0.1744 | 0.4289 | 555 | 0.41 | 0.6845 | 1.0000 | 0.05 | -0.6681 | 1.0169 | -1.2007 | 1.5495 |
| **sex m** | **1** | **9** | -0.5041 | 0.4009 | 555 | -1.26 | 0.2091 | 0.9998 | 0.05 | -1.2915 | 0.2834 | -1.7892 | 0.7811 |
| **sex m** | **2** | **3** | -0.4777 | 0.3425 | 555 | -1.39 | 0.1636 | 0.9984 | 0.05 | -1.1504 | 0.1950 | -1.5756 | 0.6202 |
| **sex m** | **2** | **4** | 1.7387 | 0.4636 | 555 | 3.75 | 0.0002 | 0.0070 | 0.05 | 0.8280 | 2.6494 | 0.2523 | 3.2251 |
| **sex m** | **2** | **5** | 1.0602 | 0.3998 | 555 | 2.65 | 0.0082 | 0.2576 | 0.05 | 0.2748 | 1.8456 | -0.2216 | 2.3421 |
| **sex m** | **2** | **6** | 0.3970 | 0.3788 | 555 | 1.05 | 0.2950 | 1.0000 | 0.05 | -0.3470 | 1.1410 | -0.8173 | 1.6113 |
| **sex m** | **2** | **7** | -0.8000 | 0.3653 | 555 | -2.19 | 0.0289 | 0.6524 | 0.05 | -1.5175 | 0.0825 | -1.9711 | 0.3710 |
| **sex m** | **2** | **8** | 0.03416 | 0.4115 | 555 | 0.08 | 0.9339 | 1.0000 | 0.05 | -0.7741 | 0.8424 | -1.2850 | 1.3533 |
| **sex m** | **2** | **9** | -0.6443 | 0.3818 | 555 | -1.69 | 0.0920 | 0.9691 | 0.05 | -1.3942 | 0.1056 | -1.8682 | 0.5797 |
| **sex m** | **3** | **4** | 2.2164 | 0.4492 | 555 | 4.93 | <.0001 | <.0001 | 0.05 | 1.3341 | 3.0986 | 0.7764 | 3.6563 |
| **sex m** | **3** | **5** | 1.5379 | 0.3825 | 555 | 4.02 | <.0001 | 0.0024 | 0.05 | 0.7866 | 2.2892 | 0.3117 | 2.7642 |
| **sex m** | **3** | **6** | 0.8747 | 0.3619 | 555 | 2.42 | 0.0160 | 0.4399 | 0.05 | 0.1638 | 1.5855 | -0.2855 | 2.0349 |
| **sex m** | **3** | **7** | -0.3224 | 0.3519 | 555 | -0.92 | 0.3600 | 1.0000 | 0.05 | -1.0135 | 0.3688 | -1.4504 | 0.8057 |
| **sex m** | **3** | **8** | 0.5118 | 0.4003 | 555 | 1.28 | 0.2016 | 0.9997 | 0.05 | -0.2745 | 1.2982 | -0.7716 | 1.7953 |
| **sex m** | **3** | **9** | -0.1666 | 0.3696 | 555 | -0.45 | 0.6523 | 1.0000 | 0.05 | -0.8926 | 0.5594 | -1.3515 | 1.0183 |
| **sex m** | **4** | **5** | -0.6784 | 0.4941 | 555 | -1.37 | 0.1703 | 0.9988 | 0.05 | -1.6491 | 0.2922 | -2.2626 | 0.9058 |
| **sex m** | **4** | **6** | -1.3417 | 0.4780 | 555 | -2.81 | 0.0052 | 0.1704 | 0.05 | -2.2806 | -0.4028 | -2.8740 | 0.1907 |
| **sex m** | **4** | **7** | -2.5387 | 0.4682 | 555 | -5.42 | <.0001 | <.0001 | 0.05 | -3.4584 | -1.6190 | -4.0398 | -1.0377 |
| **sex m** | **4** | **8** | -1.7045 | 0.5049 | 555 | -3.38 | 0.0008 | 0.0279 | 0.05 | -2.6962 | -0.7128 | -3.3231 | -0.08594 |
| **sex m** | **4** | **9** | -2.3830 | 0.4810 | 555 | -4.95 | <.0001 | <.0001 | 0.05 | -3.3278 | -1.4381 | -3.9251 | -0.8408 |
| **sex m** | **5** | **6** | -0.6632 | 0.4166 | 555 | -1.59 | 0.1119 | 0.9861 | 0.05 | -1.4815 | 0.1550 | -1.9987 | 0.6722 |
| **sex m** | **5** | **7** | -1.8603 | 0.4072 | 555 | -4.57 | <.0001 | 0.0002 | 0.05 | -2.6600 | -1.0605 | -3.1656 | -0.5550 |
| **sex m** | **5** | **8** | -1.0261 | 0.4496 | 555 | -2.28 | 0.0229 | 0.5651 | 0.05 | -1.9093 | -0.1429 | -2.4676 | 0.4154 |
| **sex m** | **5** | **9** | -1.7045 | 0.4224 | 555 | -4.04 | <.0001 | 0.0022 | 0.05 | -2.5342 | -0.8748 | -3.0587 | -0.3503 |
| **sex m** | **6** | **7** | -1.1970 | 0.3820 | 555 | -3.13 | 0.0018 | 0.0635 | 0.05 | -1.9475 | -0.4466 | -2.4218 | 0.02775 |
| **sex m** | **6** | **8** | -0.3628 | 0.4261 | 555 | -0.85 | 0.3948 | 1.0000 | 0.05 | -1.1997 | 0.4741 | -1.7288 | 1.0031 |
| **sex m** | **6** | **9** | -1.0413 | 0.3976 | 555 | -2.62 | 0.0091 | 0.2792 | 0.05 | -1.8222 | -0.2604 | -2.3158 | 0.2332 |
| **sex m** | **7** | **8** | 0.8342 | 0.3899 | 555 | 2.14 | 0.0328 | 0.6992 | 0.05 | 0.06837 | 1.6000 | -0.4157 | 2.0841 |
| **sex m** | **7** | **9** | 0.1558 | 0.3575 | 555 | 0.44 | 0.6632 | 1.0000 | 0.05 | -0.5464 | 0.8579 | -0.9902 | 1.3017 |
| **sex m** | **8** | **9** | -0.6784 | 0.3985 | 555 | -1.70 | 0.0892 | 0.9655 | 0.05 | -1.4613 | 0.1044 | -1.9561 | 0.5992 |
