# Supplementary material for: Western corn rootworm adult activity and immigrant resistance to Bt traits in first-year maize
Source: PLoS One. 2025 Jun 13;20(6):e0325388. doi: 10.1371/journal.pone.0325388 (PMC12165417; doi:10.1371/journal.pone.0325388)
Supplement: S3 Table — (DOCX) [file pone.0325388.s003.docx]

**S3 Table. Western corn rootworm adults caught on Pherocon AM unbaited sticky traps**

**by field, collection period, trap; and sex; nine collection-period dataset, 13 July – 15 September 2021 (Fd1-Fd3), 2022 (Fd 5).**

|  |  |  |  |  | **Females w/** | |  |
| --- | --- | --- | --- | --- | --- | --- | --- |
|  |  |  |  |  | **visible egg** | | |
| **Field** | **Period** | **Trap** | **Males** | **Females** | **development** |  |  |
| 1 | 1 | 1 | 3 | 1 | 0 |  |  |
| 1 | 1 | 2 | 0 | 0 | 0 |  |  |
| 1 | 1 | 3 | 2 | 1 | 0 |  |  |
| 1 | 1 | 4 | 1 | 0 | 0 |  |  |
| 1 | 1 | 5 | 4 | 0 | 0 |  |  |
| 1 | 1 | 6 | 1 | 0 | 0 |  |  |
| 1 | 1 | 7 | 0 | 1 | 0 |  |  |
| 1 | 1 | 8 | 0 | 1 | 0 |  |  |
| 1 | 2 | 1 | 0 | 1 | 0 |  |  |
| 1 | 2 | 2 | 0 | 0 | 0 |  |  |
| 1 | 2 | 3 | 0 | 0 | 0 |  |  |
| 1 | 2 | 4 | 0 | 0 | 0 |  |  |
| 1 | 2 | 5 | 0 | 0 | 0 |  |  |
| 1 | 2 | 6 | 0 | 0 | 0 |  |  |
| 1 | 2 | 7 | 0 | 0 | 0 |  |  |
| 1 | 2 | 8 | 0 | 0 | 0 |  |  |
| 1 | 3 | 1 | 0 | 0 | 0 |  |  |
| 1 | 3 | 2 | 0 | 0 | 0 |  |  |
| 1 | 3 | 3 | 0 | 0 | 0 |  |  |
| 1 | 3 | 4 | 0 | 0 | 0 |  |  |
| 1 | 3 | 5 | 0 | 0 | 0 |  |  |
| 1 | 3 | 6 | 0 | 0 | 0 |  |  |
| 1 | 3 | 7 | 0 | 0 | 0 |  |  |
| 1 | 3 | 8 | 0 | 2 | 2 |  |  |
| 1 | 4 | 1 | 0 | 0 | 0 |  |  |
| 1 | 4 | 2 | 0 | 0 | 0 |  |  |
| 1 | 4 | 3 | 0 | 0 | 0 |  |  |
| 1 | 4 | 4 | 0 | 0 | 0 |  |  |
| 1 | 4 | 5 | 0 | 0 | 0 |  |  |
| 1 | 4 | 6 | 0 | 0 | 0 |  |  |
| 1 | 4 | 7 | 0 | 0 | 0 |  |  |
| 1 | 4 | 8 | 0 | 0 | 0 |  |  |
| 1 | 5 | 1 | 0 | 0 | 0 |  |  |
| 1 | 5 | 2 | 0 | 0 | 0 |  |  |
| 1 | 5 | 3 | 0 | 0 | 0 |  |  |
| 1 | 5 | 4 | 0 | 0 | 0 |  |  |
| 1 | 5 | 5 | 0 | 0 | 0 |  |  |
| 1 | 5 | 6 | 0 | 0 | 0 |  |  |
| 1 | 5 | 7 | 0 | 1 | 1 |  |  |
| 1 | 5 | 8 | 0 | 0 | 0 |  |  |
| 1 | 6 | 1 | 0 | 0 | 0 |  |  |
| 1 | 6 | 2 | 0 | 0 | 0 |  |  |
| 1 | 6 | 3 | 0 | 0 | 0 |  |  |
| 1 | 6 | 4 | 0 | 1 | 1 |  |  |
| 1 | 6 | 5 | 0 | 0 | 0 |  |  |
| 1 | 6 | 6 | 0 | 0 | 0 |  |  |
| 1 | 6 | 7 | 0 | 0 | 0 |  |  |
| 1 | 6 | 8 | 0 | 0 | 0 |  |  |
| 1 | 7 | 1 | 0 | 0 | 0 |  |  |
| 1 | 7 | 2 | 0 | 0 | 0 |  |  |
| 1 | 7 | 3 | 1 | 0 | 0 |  |  |
| 1 | 7 | 4 | 0 | 0 | 0 |  |  |
| 1 | 7 | 5 | 1 | 0 | 0 |  |  |
| 1 | 7 | 6 | 0 | 0 | 0 |  |  |
| 1 | 7 | 7 | 0 | 0 | 0 |  |  |
| 1 | 7 | 8 | 0 | 0 | 0 |  |  |
| 1 | 8 | 1 | 0 | 0 | 0 |  |  |
| 1 | 8 | 2 | 0 | 1 | 1 |  |  |
| 1 | 8 | 3 | 0 | 0 | 0 |  |  |
| 1 | 8 | 4 | 0 | 0 | 0 |  |  |
| 1 | 8 | 5 | 0 | 0 | 0 |  |  |
| 1 | 8 | 6 | 1 | 0 | 0 |  |  |
| 1 | 8 | 7 | 2 | 0 | 0 |  |  |
| 1 | 8 | 8 | 0 | 0 | 0 |  |  |
| 1 | 9 | 1 | 1 | 0 | 0 |  |  |
| 1 | 9 | 2 | 1 | 0 | 0 |  |  |
| 1 | 9 | 3 | 0 | 0 | 0 |  |  |
| 1 | 9 | 4 | 0 | 0 | 0 |  |  |
| 1 | 9 | 5 | 0 | 0 | 0 |  |  |
| 1 | 9 | 6 | 3 | 0 | 0 |  |  |
| 1 | 9 | 7 | 0 | 0 | 0 |  |  |
| 1 | 9 | 8 | 0 | 0 | 0 |  |  |
| 2 | 1 | 1 | 0 | 1 | 0 |  |  |
| 2 | 1 | 2 | 1 | 0 | 0 |  |  |
| 2 | 1 | 3 | 1 | 1 | 0 |  |  |
| 2 | 1 | 4 | 0 | 1 | 0 |  |  |
| 2 | 1 | 5 | 0 | 0 | 0 |  |  |
| 2 | 1 | 6 | 1 | 0 | 0 |  |  |
| 2 | 1 | 7 | 0 | 0 | 0 |  |  |
| 2 | 1 | 8 | 0 | 0 | 0 |  |  |
| 2 | 2 | 1 | 0 | 3 | 1 |  |  |
| 2 | 2 | 2 | 1 | 0 | 0 |  |  |
| 2 | 2 | 3 | 1 | 0 | 0 |  |  |
| 2 | 2 | 4 | 0 | 0 | 0 |  |  |
| 2 | 2 | 5 | 1 | 0 | 0 |  |  |
| 2 | 2 | 6 | 1 | 1 | 1 |  |  |
| 2 | 2 | 7 | 1 | 2 | 0 |  |  |
| 2 | 2 | 8 | 1 | 2 | 1 |  |  |
| 2 | 3 | 1 | 0 | 1 | 0 |  |  |
| 2 | 3 | 2 | 1 | 3 | 3 |  |  |
| 2 | 3 | 3 | 0 | 2 | 2 |  |  |
| 2 | 3 | 4 | 1 | 0 | 0 |  |  |
| 2 | 3 | 5 | 1 | 0 | 0 |  |  |
| 2 | 3 | 6 | 0 | 0 | 0 |  |  |
| 2 | 3 | 7 | 0 | 0 | 0 |  |  |
| 2 | 3 | 8 | 0 | 1 | 1 |  |  |
| 2 | 4 | 1 | 0 | 0 | 0 |  |  |
| 2 | 4 | 2 | 0 | 3 | 3 |  |  |
| 2 | 4 | 3 | 0 | 1 | 0 |  |  |
| 2 | 4 | 4 | 0 | 0 | 0 |  |  |
| 2 | 4 | 5 | 0 | 1 | 1 |  |  |
| 2 | 4 | 6 | 0 | 1 | 1 |  |  |
| 2 | 4 | 7 | 0 | 2 | 2 |  |  |
| 2 | 4 | 8 | 0 | 1 | 0 |  |  |
| 2 | 5 | 1 | 0 | 0 | 0 |  |  |
| 2 | 5 | 2 | 0 | 0 | 0 |  |  |
| 2 | 5 | 3 | 0 | 1 | 1 |  |  |
| 2 | 5 | 4 | 0 | 1 | 1 |  |  |
| 2 | 5 | 5 | 0 | 1 | 1 |  |  |
| 2 | 5 | 6 | 0 | 0 | 0 |  |  |
| 2 | 5 | 7 | 0 | 1 | 1 |  |  |
| 2 | 5 | 8 | 0 | 5 | 5 |  |  |
| 2 | 6 | 1 | 0 | 1 | 1 |  |  |
| 2 | 6 | 2 | 2 | 0 | 0 |  |  |
| 2 | 6 | 3 | 2 | 3 | 3 |  |  |
| 2 | 6 | 4 | 0 | 2 | 2 |  |  |
| 2 | 6 | 5 | 0 | 1 | 1 |  |  |
| 2 | 6 | 6 | 1 | 0 | 0 |  |  |
| 2 | 6 | 7 | 0 | 0 | 0 |  |  |
| 2 | 6 | 8 | 0 | 4 | 4 |  |  |
| 2 | 7 | 1 | 5 | 3 | 3 |  |  |
| 2 | 7 | 2 | 1 | 2 | 2 |  |  |
| 2 | 7 | 3 | 2 | 1 | 1 |  |  |
| 2 | 7 | 4 | 0 | 0 | 0 |  |  |
| 2 | 7 | 5 | 2 | 1 | 1 |  |  |
| 2 | 7 | 6 | 0 | 0 | 0 |  |  |
| 2 | 7 | 7 | 0 | 0 | 0 |  |  |
| 2 | 7 | 8 | 0 | 0 | 0 |  |  |
| 2 | 8 | 1 | 2 | 0 | 0 |  |  |
| 2 | 8 | 2 | 0 | 0 | 0 |  |  |
| 2 | 8 | 3 | 0 | 0 | 0 |  |  |
| 2 | 8 | 4 | 0 | 0 | 0 |  |  |
| 2 | 8 | 5 | 0 | 0 | 0 |  |  |
| 2 | 8 | 6 | 0 | 0 | 0 |  |  |
| 2 | 8 | 7 | 0 | 0 | 0 |  |  |
| 2 | 8 | 8 | 0 | 0 | 0 |  |  |
| 2 | 9 | 1 | 2 | 0 | 0 |  |  |
| 2 | 9 | 2 | 0 | 0 | 0 |  |  |
| 2 | 9 | 3 | 0 | 0 | 0 |  |  |
| 2 | 9 | 4 | 1 | 0 | 0 |  |  |
| 2 | 9 | 5 | 0 | 0 | 0 |  |  |
| 2 | 9 | 6 | 0 | 0 | 0 |  |  |
| 2 | 9 | 7 | 0 | 0 | 0 |  |  |
| 2 | 9 | 8 | 0 | 0 | 0 |  |  |
| 3 | 1 | 1 | 0 | 0 | 0 |  |  |
| 3 | 1 | 2 | 1 | 0 | 0 |  |  |
| 3 | 1 | 3 | 0 | 0 | 0 |  |  |
| 3 | 1 | 4 | 1 | 0 | 0 |  |  |
| 3 | 1 | 5 | 0 | 0 | 0 |  |  |
| 3 | 1 | 6 | 0 | 0 | 0 |  |  |
| 3 | 1 | 7 | 0 | 0 | 0 |  |  |
| 3 | 1 | 8 | 0 | 0 | 0 |  |  |
| 3 | 2 | 1 | 0 | 0 | 0 |  |  |
| 3 | 2 | 2 | 0 | 0 | 0 |  |  |
| 3 | 2 | 3 | 0 | 0 | 0 |  |  |
| 3 | 2 | 4 | 1 | 0 | 0 |  |  |
| 3 | 2 | 5 | 0 | 0 | 0 |  |  |
| 3 | 2 | 6 | 0 | 0 | 0 |  |  |
| 3 | 2 | 7 | 0 | 0 | 0 |  |  |
| 3 | 2 | 8 | 0 | 0 | 0 |  |  |
| 3 | 3 | 1 | 0 | 0 | 0 |  |  |
| 3 | 3 | 2 | 0 | 0 | 0 |  |  |
| 3 | 3 | 3 | 0 | 0 | 0 |  |  |
| 3 | 3 | 4 | 0 | 0 | 0 |  |  |
| 3 | 3 | 5 | 0 | 0 | 0 |  |  |
| 3 | 3 | 6 | 0 | 0 | 0 |  |  |
| 3 | 3 | 7 | 0 | 0 | 0 |  |  |
| 3 | 3 | 8 | 0 | 0 | 0 |  |  |
| 3 | 4 | 1 | 0 | 0 | 0 |  |  |
| 3 | 4 | 2 | 0 | 0 | 0 |  |  |
| 3 | 4 | 3 | 0 | 0 | 0 |  |  |
| 3 | 4 | 4 | 0 | 0 | 0 |  |  |
| 3 | 4 | 5 | 0 | 0 | 0 |  |  |
| 3 | 4 | 6 | 0 | 0 | 0 |  |  |
| 3 | 4 | 7 | 0 | 0 | 0 |  |  |
| 3 | 4 | 8 | 1 | 0 | 0 |  |  |
| 3 | 5 | 1 | 0 | 0 | 0 |  |  |
| 3 | 5 | 2 | 0 | 0 | 0 |  |  |
| 3 | 5 | 3 | 0 | 0 | 0 |  |  |
| 3 | 5 | 4 | 0 | 1 | 1 |  |  |
| 3 | 5 | 5 | 0 | 0 | 0 |  |  |
| 3 | 5 | 6 | 0 | 0 | 0 |  |  |
| 3 | 5 | 7 | 0 | 0 | 0 |  |  |
| 3 | 5 | 8 | 0 | 1 | 1 |  |  |
| 3 | 6 | 1 | 0 | 0 | 0 |  |  |
| 3 | 6 | 2 | 0 | 0 | 0 |  |  |
| 3 | 6 | 3 | 1 | 0 | 0 |  |  |
| 3 | 6 | 4 | 0 | 0 | 0 |  |  |
| 3 | 6 | 5 | 0 | 0 | 0 |  |  |
| 3 | 6 | 6 | 0 | 0 | 0 |  |  |
| 3 | 6 | 7 | 0 | 0 | 0 |  |  |
| 3 | 6 | 8 | 0 | 0 | 0 |  |  |
| 3 | 7 | 1 | 2 | 2 | 2 |  |  |
| 3 | 7 | 2 | 3 | 6 | 6 |  |  |
| 3 | 7 | 3 | 4 | 4 | 4 |  |  |
| 3 | 7 | 4 | 5 | 2 | 2 |  |  |
| 3 | 7 | 5 | 9 | 4 | 4 |  |  |
| 3 | 7 | 6 | 4 | 5 | 5 |  |  |
| 3 | 7 | 7 | 8 | 5 | 5 |  |  |
| 3 | 7 | 8 | 2 | 2 | 2 |  |  |
| 3 | 8 | 1 | 4 | 0 | 0 |  |  |
| 3 | 8 | 2 | 2 | 0 | 0 |  |  |
| 3 | 8 | 3 | 2 | 4 | 4 |  |  |
| 3 | 8 | 4 | 2 | 0 | 0 |  |  |
| 3 | 8 | 5 | 1 | 1 | 1 |  |  |
| 3 | 8 | 6 | 5 | 0 | 0 |  |  |
| 3 | 8 | 7 | 2 | 0 | 0 |  |  |
| 3 | 8 | 8 | 2 | 0 | 0 |  |  |
| 3 | 9 | 1 | 7 | 0 | 0 |  |  |
| 3 | 9 | 2 | 0 | 1 | 1 |  |  |
| 3 | 9 | 3 | 2 | 0 | 0 |  |  |
| 3 | 9 | 4 | 2 | 0 | 0 |  |  |
| 3 | 9 | 5 | 11 | 4 | 4 |  |  |
| 3 | 9 | 6 | 5 | 0 | 0 |  |  |
| 3 | 9 | 7 | 7 | 0 | 0 |  |  |
| 3 | 9 | 8 | 3 | 1 | 1 |  |  |
| 5 | 1 | 1 | 3 | 0 | 0 |  |  |
| 5 | 1 | 2 | 7 | 0 | 0 |  |  |
| 5 | 1 | 3 | 3 | 1 | 0 |  |  |
| 5 | 1 | 4 | 5 | 0 | 0 |  |  |
| 5 | 1 | 5 | 5 | 0 | 0 |  |  |
| 5 | 1 | 6 | 8 | 0 | 0 |  |  |
| 5 | 1 | 7 | 5 | 3 | 0 |  |  |
| 5 | 1 | 8 | 2 | 0 | 0 |  |  |
| 5 | 2 | 1 | 8 | 4 | 2 |  |  |
| 5 | 2 | 2 | 3 | 3 | 3 |  |  |
| 5 | 2 | 3 | 24 | 3 | 3 |  |  |
| 5 | 2 | 4 | 22 | 4 | 4 |  |  |
| 5 | 2 | 5 | 10 | 2 | 1 |  |  |
| 5 | 2 | 6 | 6 | 2 | 2 |  |  |
| 5 | 2 | 7 | 3 | 4 | 4 |  |  |
| 5 | 2 | 8 | 16 | 3 | 3 |  |  |
| 5 | 3 | 1 | 45 | 7 | 7 |  |  |
| 5 | 3 | 2 | 13 | 5 | 5 |  |  |
| 5 | 3 | 3 | 55 | 7 | 7 |  |  |
| 5 | 3 | 4 | 36 | 6 | 6 |  |  |
| 5 | 3 | 5 | 9 | 6 | 6 |  |  |
| 5 | 3 | 6 | 15 | 4 | 4 |  |  |
| 5 | 3 | 7 | 29 | 10 | 10 |  |  |
| 5 | 3 | 8 | 12 | 7 | 7 |  |  |
| 5 | 4 | 1 | 3 | 3 | 3 |  |  |
| 5 | 4 | 2 | 0 | 2 | 2 |  |  |
| 5 | 4 | 3 | 2 | 2 | 2 |  |  |
| 5 | 4 | 4 | 3 | 3 | 3 |  |  |
| 5 | 4 | 5 | 3 | 7 | 7 |  |  |
| 5 | 4 | 6 | 2 | 6 | 6 |  |  |
| 5 | 4 | 7 | 0 | 1 | 1 |  |  |
| 5 | 4 | 8 | 0 | 0 | 0 |  |  |
| 5 | 5 | 1 | 5 | 3 | 3 |  |  |
| 5 | 5 | 2 | 3 | 8 | 8 |  |  |
| 5 | 5 | 3 | 2 | 4 | 4 |  |  |
| 5 | 5 | 4 | 3 | 7 | 7 |  |  |
| 5 | 5 | 5 | 8 | 22 | 22 |  |  |
| 5 | 5 | 6 | 10 | 15 | 15 |  |  |
| 5 | 5 | 7 | 1 | 9 | 9 |  |  |
| 5 | 5 | 8 | 2 | 3 | 3 |  |  |
| 5 | 6 | 1 | 10 | 20 | 20 |  |  |
| 5 | 6 | 2 | 8 | 14 | 14 |  |  |
| 5 | 6 | 3 | 6 | 11 | 11 |  |  |
| 5 | 6 | 4 | 10 | 38 | 38 |  |  |
| 5 | 6 | 5 | 6 | 30 | 30 |  |  |
| 5 | 6 | 6 | 4 | 18 | 18 |  |  |
| 5 | 6 | 7 | 6 | 10 | 10 |  |  |
| 5 | 6 | 8 | 2 | 7 | 7 |  |  |
| 5 | 7 | 1 | 3 | 14 | 14 |  |  |
| 5 | 7 | 2 | 3 | 20 | 20 |  |  |
| 5 | 7 | 3 | 3 | 26 | 26 |  |  |
| 5 | 7 | 4 | 10 | 55 | 55 |  |  |
| 5 | 7 | 5 | 2 | 24 | 24 |  |  |
| 5 | 7 | 6 | 3 | 22 | 22 |  |  |
| 5 | 7 | 7 | 1 | 16 | 16 |  |  |
| 5 | 7 | 8 | 6 | 27 | 27 |  |  |
| 5 | 8 | 1 | 0 | 4 | 4 |  |  |
| 5 | 8 | 2 | 0 | 1 | 1 |  |  |
| 5 | 8 | 3 | 0 | 2 | 2 |  |  |
| 5 | 8 | 4 | 0 | 4 | 4 |  |  |
| 5 | 8 | 5 | 0 | 2 | 2 |  |  |
| 5 | 8 | 6 | 0 | 0 | 0 |  |  |
| 5 | 8 | 7 | 0 | 1 | 1 |  |  |
| 5 | 8 | 8 | 0 | 0 | 0 |  |  |
| 5 | 9 | 1 | 0 | 2 | 2 |  |  |
| 5 | 9 | 2 | 0 | 2 | 2 |  |  |
| 5 | 9 | 3 | 0 | 1 | 1 |  |  |
| 5 | 9 | 4 | 0 | 3 | 3 |  |  |
| 5 | 9 | 5 | 0 | 0 | 0 |  |  |
| 5 | 9 | 6 | 0 | 1 | 1 |  |  |
| 5 | 9 | 7 | 0 | 0 | 0 |  |  |
| 5 | 9 | 8 | 0 | 0 | 0 |  |  |
