# Supplementary material for: Western corn rootworm adult activity and immigrant resistance to Bt traits in first-year maize
Source: PLoS One. 2025 Jun 13;20(6):e0325388. doi: 10.1371/journal.pone.0325388 (PMC12165417; doi:10.1371/journal.pone.0325388)
Supplement: S4 Table — (DOCX) [file pone.0325388.s004.docx]

**S4 Table. Western corn rootworm adults caught on Pherocon AM unbaited sticky traps**

**by field, collection period, trap, and sex; six collection-period dataset, 14 July – 25 August 2022.**

| **Field** | **Period** | **Trap** | **Males** | **Females** | **Females w/visible egg development** |
| --- | --- | --- | --- | --- | --- |
| 6 | 1 | 1 | 13 | 1 | 0 |
| 6 | 1 | 2 | 7 | 1 | 0 |
| 6 | 1 | 3 | 1 | 0 | 0 |
| 6 | 1 | 4 | 6 | 1 | 0 |
| 6 | 1 | 5 | 9 | 0 | 0 |
| 6 | 1 | 6 | 16 | 1 | 0 |
| 6 | 1 | 7 | 7 | 0 | 0 |
| 6 | 1 | 8 | 13 | 0 | 0 |
| 6 | 2 | 1 | 6 | 3 | 3 |
| 6 | 2 | 2 | 1 | 4 | 4 |
| 6 | 2 | 3 | 1 | 2 | 2 |
| 6 | 2 | 4 | 5 | 1 | 1 |
| 6 | 2 | 5 | 11 | 4 | 3 |
| 6 | 2 | 6 | 9 | 2 | 2 |
| 6 | 2 | 7 | 4 | 3 | 3 |
| 6 | 2 | 8 | 14 | 2 | 2 |
| 6 | 3 | 1 | 0 | 3 | 3 |
| 6 | 3 | 2 | 0 | 0 | 0 |
| 6 | 3 | 3 | 1 | 0 | 0 |
| 6 | 3 | 4 | 0 | 0 | 0 |
| 6 | 3 | 5 | 4 | 2 | 2 |
| 6 | 3 | 6 | 5 | 1 | 1 |
| 6 | 3 | 7 | 3 | 2 | 2 |
| 6 | 3 | 8 | 5 | 2 | 2 |
| 6 | 4 | 1 | 2 | 2 | 2 |
| 6 | 4 | 2 | 0 | 0 | 0 |
| 6 | 4 | 3 | 0 | 1 | 1 |
| 6 | 4 | 4 | 1 | 3 | 3 |
| 6 | 4 | 5 | 1 | 5 | 5 |
| 6 | 4 | 6 | 1 | 6 | 6 |
| 6 | 4 | 7 | 2 | 2 | 2 |
| 6 | 4 | 8 | 0 | 2 | 2 |
| 6 | 5 | 1 | 4 | 1 | 1 |
| 6 | 5 | 2 | 2 | 0 | 0 |
| 6 | 5 | 3 | 0 | 3 | 3 |
| 6 | 5 | 4 | 1 | 2 | 2 |
| 6 | 5 | 5 | 2 | 4 | 4 |
| 6 | 5 | 6 | 3 | 6 | 6 |
| 6 | 5 | 7 | 1 | 7 | 7 |
| 6 | 5 | 8 | 1 | 3 | 3 |
| 6 | 6 | 1 | 0 | 1 | 1 |
| 6 | 6 | 2 | 0 | 0 | 0 |
| 6 | 6 | 3 | 0 | 1 | 1 |
| 6 | 6 | 4 | 0 | 2 | 2 |
| 6 | 6 | 5 | 0 | 4 | 4 |
| 6 | 6 | 6 | 0 | 3 | 3 |
| 6 | 6 | 7 | 0 | 5 | 5 |
| 6 | 6 | 8 | 0 | 3 | 3 |
| 7 | 1 | 1 | 0 | 0 | 0 |
| 7 | 1 | 2 | 2 | 0 | 0 |
| 7 | 1 | 3 | 2 | 0 | 0 |
| 7 | 1 | 4 | 1 | 0 | 0 |
| 7 | 1 | 5 | 1 | 0 | 0 |
| 7 | 1 | 6 | 0 | 0 | 0 |
| 7 | 1 | 7 | 4 | 0 | 0 |
| 7 | 1 | 8 | 2 | 0 | 0 |
| 7 | 2 | 1 | 0 | 0 | 0 |
| 7 | 2 | 2 | 0 | 0 | 0 |
| 7 | 2 | 3 | 1 | 0 | 0 |
| 7 | 2 | 4 | 1 | 0 | 0 |
| 7 | 2 | 5 | 3 | 0 | 0 |
| 7 | 2 | 6 | 5 | 0 | 0 |
| 7 | 2 | 7 | 7 | 2 | 2 |
| 7 | 2 | 8 | 11 | 3 | 3 |
| 7 | 3 | 1 | 3 | 0 | 0 |
| 7 | 3 | 2 | 2 | 0 | 0 |
| 7 | 3 | 3 | 2 | 0 | 0 |
| 7 | 3 | 4 | 0 | 0 | 0 |
| 7 | 3 | 5 | 2 | 1 | 1 |
| 7 | 3 | 6 | 1 | 3 | 3 |
| 7 | 3 | 7 | 2 | 2 | 2 |
| 7 | 3 | 8 | 4 | 4 | 4 |
| 7 | 4 | 1 | 1 | 2 | 2 |
| 7 | 4 | 2 | 0 | 1 | 1 |
| 7 | 4 | 3 | 0 | 2 | 2 |
| 7 | 4 | 4 | 0 | 0 | 0 |
| 7 | 4 | 5 | 0 | 1 | 1 |
| 7 | 4 | 6 | 0 | 0 | 0 |
| 7 | 4 | 7 | 2 | 3 | 3 |
| 7 | 4 | 8 | 0 | 1 | 1 |
| 7 | 5 | 1 | 0 | 1 | 1 |
| 7 | 5 | 2 | 0 | 3 | 3 |
| 7 | 5 | 3 | 0 | 0 | 0 |
| 7 | 5 | 4 | 0 | 2 | 2 |
| 7 | 5 | 5 | 0 | 0 | 0 |
| 7 | 5 | 6 | 0 | 1 | 1 |
| 7 | 5 | 7 | 0 | 1 | 1 |
| 7 | 5 | 8 | 0 | 1 | 1 |
| 7 | 6 | 1 | 0 | 1 | 1 |
| 7 | 6 | 2 | 0 | 0 | 0 |
| 7 | 6 | 3 | 0 | 0 | 0 |
| 7 | 6 | 4 | 0 | 0 | 0 |
| 7 | 6 | 5 | 0 | 1 | 1 |
| 7 | 6 | 6 | 0 | 0 | 0 |
| 7 | 6 | 7 | 0 | 0 | 0 |
| 7 | 6 | 8 | 1 | 1 | 1 |
| 8 | 1 | 1 | 2 | 1 | 0 |
| 8 | 1 | 2 | 4 | 0 | 0 |
| 8 | 1 | 3 | 1 | 0 | 0 |
| 8 | 1 | 4 | 3 | 0 | 0 |
| 8 | 1 | 5 | 3 | 0 | 0 |
| 8 | 1 | 6 | 2 | 0 | 0 |
| 8 | 1 | 7 | 1 | 0 | 0 |
| 8 | 1 | 8 | 2 | 0 | 0 |
| 8 | 2 | 1 | 8 | 5 | 5 |
| 8 | 2 | 2 | 4 | 3 | 3 |
| 8 | 2 | 3 | 3 | 1 | 1 |
| 8 | 2 | 4 | 3 | 3 | 3 |
| 8 | 2 | 5 | 5 | 2 | 2 |
| 8 | 2 | 6 | 1 | 2 | 2 |
| 8 | 2 | 7 | 1 | 2 | 2 |
| 8 | 2 | 8 | 0 | 6 | 6 |
| 8 | 3 | 1 | 4 | 3 | 3 |
| 8 | 3 | 2 | 3 | 0 | 0 |
| 8 | 3 | 3 | 3 | 0 | 0 |
| 8 | 3 | 4 | 3 | 1 | 1 |
| 8 | 3 | 5 | 1 | 0 | 0 |
| 8 | 3 | 6 | 2 | 1 | 1 |
| 8 | 3 | 7 | 1 | 1 | 1 |
| 8 | 3 | 8 | 3 | 2 | 2 |
| 8 | 4 | 1 | 0 | 0 | 0 |
| 8 | 4 | 2 | 0 | 0 | 0 |
| 8 | 4 | 3 | 0 | 0 | 0 |
| 8 | 4 | 4 | 0 | 1 | 1 |
| 8 | 4 | 5 | 0 | 1 | 1 |
| 8 | 4 | 6 | 0 | 0 | 0 |
| 8 | 4 | 7 | 0 | 0 | 0 |
| 8 | 4 | 8 | 0 | 3 | 3 |
| 8 | 5 | 1 | 0 | 2 | 2 |
| 8 | 5 | 2 | 0 | 2 | 2 |
| 8 | 5 | 3 | 1 | 2 | 2 |
| 8 | 5 | 4 | 0 | 0 | 0 |
| 8 | 5 | 5 | 0 | 0 | 0 |
| 8 | 5 | 6 | 0 | 2 | 2 |
| 8 | 5 | 7 | 0 | 2 | 2 |
| 8 | 5 | 8 | 0 | 1 | 1 |
| 8 | 6 | 1 | 1 | 0 | 0 |
| 8 | 6 | 2 | 0 | 2 | 2 |
| 8 | 6 | 3 | 0 | 2 | 2 |
| 8 | 6 | 4 | 3 | 5 | 5 |
| 8 | 6 | 5 | 0 | 2 | 2 |
| 8 | 6 | 6 | 0 | 5 | 5 |
| 8 | 6 | 7 | 2 | 1 | 1 |
| 8 | 6 | 8 | 1 | 6 | 6 |
